# ZO-1/Tjp1 and ZO-2/Tjp2 deletion in retinal pigment epithelium causes progressive retinal degeneration

**DOI:** 10.1016/j.isci.2025.113673

**Published:** 2025-10-03

**Authors:** Safiah Mohamed Ali, Bhav Harshad Parikh, Queenie Shu Woon Tan, Barbara Hübner, Aleksandra N. Kozyrina, Animesh Banerjee, Jie Zhao, Debbie Goh, Hanumakumar Bogireddi, Sia Wey Yeo, Daniel Soo Lin Wong, Jianliang Xu, Kim Chi Tran, Zengping Liu, Yun-Zheng Le, Veluchamy Amutha Barathi, Kang Hao Cheong, Jacopo Di Russo, Alexander Ludwig, Walter Hunziker, Xinyi Su

**Affiliations:** 1 Institute of Molecular and Cell Biology (IMCB), Agency for Science, Technology and Research (A∗STAR), Singapore 138673, Singapore; 2 Department of Ophthalmology, Yong Loo Lin School of Medicine, National University of Singapore (NUS), Singapore 119228, Singapore; 3 School of Biological Sciences, Nanyang Technological University (NTU), Singapore 637551, Singapore; 4 NTU Institute of Structural Biology, Nanyang Technological University (NTU), Singapore 636921, Singapore; 5 Interdisciplinary Centre for Clinical Research, RWTH Aachen University, 52074 Aachen, Germany; 6 Institute of Molecular and Cellular Anatomy, RWTH Aachen University, 52074 Aachen, Germany; 7 Division of Mathematical Sciences, School of Physical and Mathematical Sciences, Nanyang Technological University (NTU), Singapore 637371, Singapore; 8 Science, Mathematics and Technology, Singapore University of Technology and Design (SUTD), Singapore 487372, Singapore; 9 Singapore Eye Research Institute (SERI), Singapore 169856, Singapore; 10 Department of Medicine, Cell Biology, and Ophthalmology and Harold Hamm Diabetes Center, University of Oklahoma Health Sciences Center, Oklahoma City OK 73104, USA; 11 Academic Clinical Program, Duke-NUS Medical School, National University of Singapore, Singapore 169857, Singapore; 12 College of Computing and Data Science, Nanyang Technological University (NTU), Singapore 639798, Singapore; 13 DWI – Leibniz-Institute for Interactive Materials, 52074 Aachen, Germany; 14 Department of Physiology, Yong Loo Lin School of Medicine, National University of Singapore (NUS), Singapore 117593, Singapore; 15 Department of Ophthalmology, National University Hospital (NUH), Singapore 119228, Singapore; 16 Centre for Innovation and Precision Eye Health, Yong Loo Lin School of Medicine, National University of Singapore (NUS), Singapore 119228, Singapore

**Keywords:** Cell biology, Model organism

## Abstract

Tight junction protein-1 and -2 (Tjp1/ZO-1 and Tjp2/ZO-2) function as scaffold proteins within the tight junction complexes of the blood-retinal barrier (BRB). Although the breakdown of the BRB is implicated in retinopathies, the contribution of ZO-1/2 in the pathogenesis of retinopathies is unknown. To understand their role, we generated RPE-specific conditional ZO-1/2 single KOs (T1KO/T2KO) and ZO-1/2 double knockout (DKO) mice. While T1KO and T2KO did not exhibit overt retinal phenotypes, DKO demonstrated a strong retinal phenotype from 1-month post-KO induction. This includes the loss of RPE integrity and retinal thinning. Furthermore, RPE in DKO re-entered the cell cycle with upregulated YAP. At 12 months, we observed severe structural and functional retinal deterioration. In response to laser-induced damage, RPE displayed persistent hyper-proliferation, delayed wound repair, and the up-regulation of YAP. These studies confirmed the critical role of ZO-1/2 in maintaining an intact BRB and revealed a role of ZO-1/2 in wound healing.

## Highlights

• Tjp1-2 double KO (DKO) disrupts RPE structure and function
• DKO RPE shows YAP upregulation and re-entry into the cell cycle
• DKO mice exhibit impaired wound healing after laser-induced damage
• DKO mice show progressive structural and functional retinal deterioration

## Graphical abstract

**Figure:**
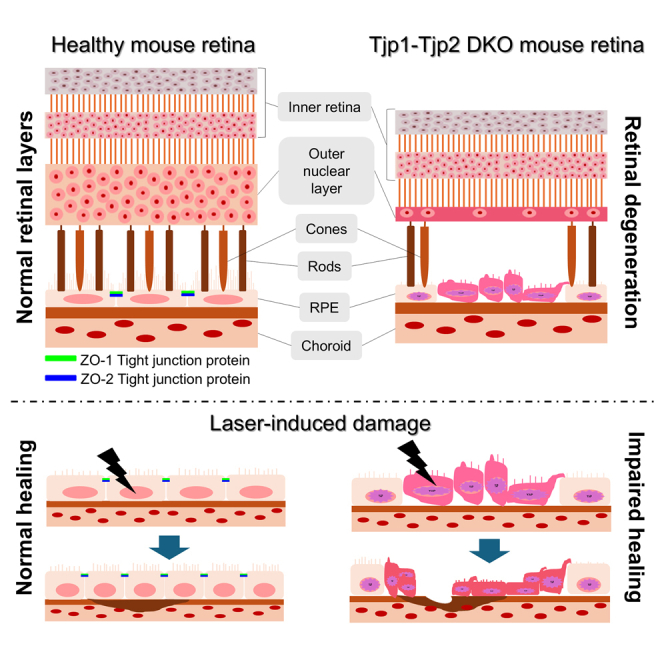

## Introduction

The retinal pigment epithelium (RPE) is a monolayer of polarized pigmented epithelial cells, which performs numerous specialized functions pivotal to retinal homeostasis.^1^ Central to the RPE function is the presence of specialized junctional complexes such as tight junctions (TJs), adherens junctions (AJs), and gap junctions. TJs in particular play a key role in the outer blood retinal barrier (oBRB) through the maintenance of RPE cell polarity and regulation of the paracellular diffusion of solutes. The loss of RPE polarity and oBRB has been associated with the development of both degenerative and proliferative retinal disorders.^2^ For example, in neovascular age-related macular degeneration (nAMD), loss of RPE TJs or cell-cell adhesion has been shown to induce vascular endothelial growth factor (VEGF) overexpression and initiate neovascularization.^3^ Moreover, in proliferative vitreoretinopathy (PVR), loss of RPE cell-cell junctions leads to epithelial-mesenchymal transition (EMT) of RPE cells, resulting in hyper-proliferation and the formation of fibrocellular membranes.^4^^,^^5^^,^^6^ Surprisingly, both these retinal diseases are influenced by YAP, a major regulator of the Hippo pathway, which is also known to associate with TJ components, including ZO-1 and ZO-2.^7^^,^^8^^,^^9^ However, it remains unclear whether the breakdown of oBRB is a cause or consequence of retinal disease progression.

Given that ZO-1 and ZO-2 knock-out results in embryonic lethality,^10^^,^^11^ most studies studying ZO-1 and ZO-2 function are conducted either *in vitro* or using conditional KO mouse models. *In vitro* knockout/silencing studies using mouse and canine kidney epithelial cells suggest some degree of redundancy of ZO-1 and ZO-2 in regulating TJ structure and function.^12^^,^^13^ While *in vivo* studies have highlighted that ZO-1 and ZO-2 have some non-redundant and independent roles leading to embryonic lethality, ZO-3 knockout mice do not show an overt phenotype.^10^^,^^14^ *In vivo* studies suggest that the ZO family of proteins shares an intricate relationship to perform particular cellular functions, as observed across different cellular contexts.^6^^,^^15^^,^^16^ Particularly in the mice retina, lentiviral mediated the inhibition of ZO-1 coupled with the upregulation of ZONAB (ZO-1-associated nucleic-acid-binding protein) affected RPE homeostasis.^15^ Although this conditional *in vivo* knockdown indicates that ZO-1 has an integral role in forming the oBRB, the role of ZO-2 in the eye and the relative contributions of both ZO proteins in retinal disease progression remain poorly understood.

To address the aforementioned questions, we generated RPE-specific single and double conditional KOs (T1KO, T2KO, and T1/T2 DKO), using a doxycycline (Dox) inducible Cre-Lox system to circumvent the embryonic lethality of ZO-1 and ZO-2 knockouts. This allowed us to perform phenotypic and molecular characterization of single and DKO mice retina, as well as examine the impact of ZO-1/2 knockout on post-laser injury, to study RPE wound healing.

## Results

### Retinal pigment epithelium-specific conditional deletion of *Tjp1* and/or *Tjp2* in mice

**Figure 1:**
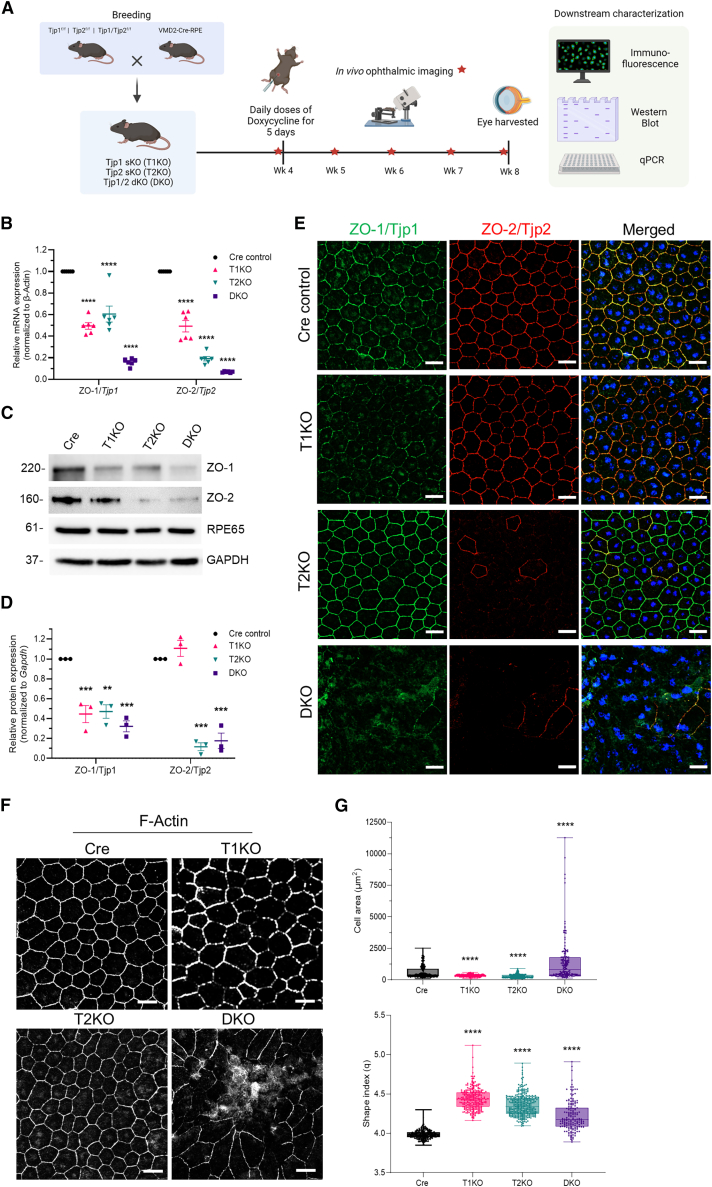
Characterization of *Vmd2*-Cre activated conditional deletions of *Tjp1* and/or *Tjp2* genes in mice RPE (A) Schematic of the generation and characterization of the 3 knockout mouse models (T1KO, T2KO, and DKO) post 1-month Dox induction. (B) Quantification of mRNA expression of *Tjp1* and *Tjp2* of all three KO genotypes compared against control Cre mice (*n* = 6 eyes per genotype), and normalized to housekeeping gene, β-Actin. (C) Deletion of the respective *Tjp* genes was confirmed by the Western blotting of RPE lysates from the respective genotypes (*n* = 3 eyes per genotype). RPE65 was used as a marker of RPE tissue, and GAPDH as a housekeeping gene. (D) Quantification of protein expression in (C) after normalization to housekeeping protein, GAPDH. (E) Expression and localization of ZO-1/Tjp1 and ZO-2/Tjp2 were analyzed using immunofluorescence (IF) in three KO mouse models and a Cre control. (F) F-actin was used to mark the cell borders of RPE in the flatmount for analysis of cell morphometry. (G) Cell area (um^2^) and Shape index (q) of RPE cells in all four mouse genotypes (*n* ≥ 3 eyes per genotype and *n* ≥ 100 cells per eye) were quantified. Data for (B), (D), and (G) are represented by mean ± SEM. Scale bar for (E) and (F), 20 μm. Statistical analyses were done comparing T1KO, T2KO, and DKO against Cre control. For (B) and (D), one-way ANOVA was performed, followed by Tukey’s honest significance difference (HSD) *post hoc* test; for (G) Kruskal-Wallis test was performed with Dunn’s multiple comparison test. (∗) = *p* < 0.05, (∗∗) = *p* < 0.01, (∗∗∗) = *p* < 0.001, (∗∗∗∗) = *p* < 0.0001.

To assess the role of the ZO-1/Tjp1 and ZO-2/Tjp2 proteins in the mouse RPE, we generated gene-specific conditional knockout mice using the Cre-LoxP system (Figure 1A). To delete *Tjp1* (gene encoding ZO-1) and/or *Tjp2* (gene encoding ZO-2) in the mouse RPE – (i) *Tjp1*^*F/F*^; (ii) *Tjp2* ^*F/F*^; (iii) *Tjp1* ^*F/*F^/Tjp2 ^*F/F*^ floxed mice were crossed with a tetracycline-inducible Cre mouse.^17^^,^^18^ Tetracycline expression was placed under the control of a human vitelliform macular dystrophy-2 (*VMD2*) promoter that is expressed exclusively in RPE.^19^ Using a Dox induction protocol on 1-month-old mice of various genotypes, we generated single knockouts (ZO-1/Tjp1 KO: T1KO and ZO-2/Tjp2 KO: T2KO) and a Tjp1/Tjp2 double knockout (DKO) mouse model. ZO-1 and ZO-2 proteins typically localize to the apical region of RPE and demarcate the polygonal structure of the post-mitotic RPE.^20^ To validate the knockout efficiency, we quantified individual *Tjp1* and *Tjp2* transcripts using RT-qPCR and observed >80% (*p* < 0.0001) reduction in both *Tjp1* and *Tjp2* transcript expression in RPE from DKO mice compared to RPE from control Cre mice (Figure 1B). In addition, we also quantified ZO-1 and ZO-2 deletion by the Western blotting of isolated RPE from respective mouse strains. We observed a reduction of at least 70% (*p* < 0.001) for both ZO-1 and ZO-2 in the RPE from DKO mice (Figures 1C and 1D).

To further visualize ZO-1 and ZO-2 deletion and its impact on RPE cell-cell junctions and cell morphology (i.e., polygonal arrangement), we performed immunofluorescence experiments on mouse RPE flat mounts. As expected, T1KO and T2KO mice RPE showed loss of only ZO-1 and ZO-2 proteins, respectively, whilst DKO RPE displayed loss of both ZO-1/2 proteins (Figure 1E). To analyze the impact on RPE tight junctions and adherens junctions, we visualized Claudin-2 and E-Cadherin, respectively. Only DKO RPE, but not T1KO and T2KO mice, displayed loss of Claudin-2, confirming the disruption of tight junctions (Figure S1). By visualizing E-Cadherin, we observed significantly increased the expression of the protein in DKO RPE flatmounts as compared to the other genotypes, indicating a potential alteration in adherens junction (Figure S2).

To quantitatively evaluate the changes in morphometric features, we stained the RPE flatmounts with F-actin (i.e., Cortical actin) and performed cell area and shape index analyses (Figure 1F). While cell area was significantly different for T1KO and T2KO against Cre control, DKO RPE demonstrated a larger increase (Figure 1G). Whereas for shape index changes, all 3 KO genotypes showed a difference as compared to the Cre control. Together, although T1KO and T2KO present differences in cell morphometry, only the DKO showed severe RPE dysmorphia and cell-cell junctional alterations – an early sign of RPE degeneration^21^^,^^22^ in the DKO mice. In summary, we show that the combined loss of ZO-1 and ZO-2 in mice RPE leads to an observable structural abnormality of the mice RPE.

### Loss of zonula occludens-1 and zonula occludens-2 in retinal pigment epithelium leads to outer blood retinal barrier dysfunction

**Figure 2:**
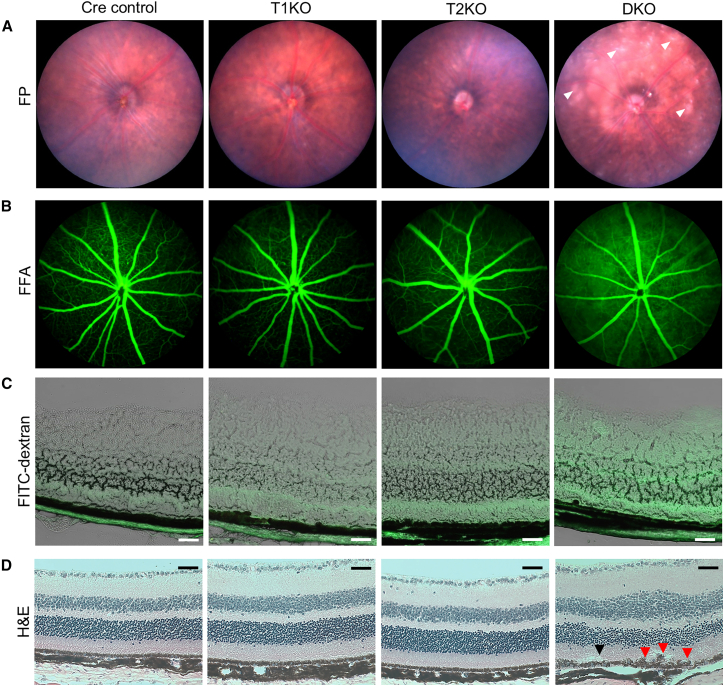
Phenotypic changes to the posterior segment, oBRB integrity, and retinal architecture at 1-month post-Dox induction (A) The posterior eye segment was visualized using fundus photography (FP). White amorphous lesions were observed only in DKO mice's eyes (white arrowheads). (B) Retinal vasculature was observed using fundus fluorescein angiography (FFA). Increased fluorescence intensity was observed in DKO mice eyes. (C) oBRB integrity was monitored using 40 kDa FITC-dextran leakage (green) from choriocapillaris to the neural retina through brightfield imaging. (D) The *ex vivo* cross-sections of the retina were visualized using H&E. It demonstrated irregularities in the RPE monolayer only in DKO RPE (red arrowheads), with possible signs of sub-retinal fluid accumulation (black arrowhead). Three independent experiments were performed for (A–D), and single representative images are shown for each. Scale bar for (C) and (D), 50 μm.

Given the overt phenotype in DKO mice, we focused next on the characterization of the DKO mice retina. Standard ophthalmic examination was performed at 1-month post-Dox induced ZO-1 and ZO-2 deletion (i.e., 2-month-old mice). Fundus photography (FP) of DKO revealed the presence of whitish amorphous lesions throughout the posterior pole, which were otherwise absent in Cre control and single KO mice (Figure 2A). This suggests the presence of underlying RPE dysfunction specifically in DKO mice.^23^ As TJs are crucial for maintaining oBRB, we interrogated retinal vascular integrity using fundus fluorescein angiography (FFA). Specifically, in DKO eyes, but not in the single KOs, we observed early phase hyper-fluorescence in the retina (Figure 2B). This is consistent with a compromised oBRB, allowing fluorescein leakage from the choriocapillaris into the retinal layers. This is typically not observed in healthy eyes, where the continuous and intact RPE monolayers mask the background hyper-fluorescence arising from intra-peritoneally injected fluorescein.^24^ To further confirm the loss of oBRB in DKO mice, we performed an *in vivo* permeability assay through the retro-orbital injection of a 40 kDa fluorescein isothiocyanate (FITC)-conjugated dextran tracer.^25^ Consistent with a disrupted oBRB, leakage of FITC-dextran from the adjacent choriocapillaris into the retina was significantly increased in DKO mice retina compared to all the other genotypes (Figures 2C and S3). Hematoxylin and eosin (H&E) staining of retina cross-sections also showed discontinuities in the RPE layer and possible signs of subretinal fluid accumulation further supporting the RPE dysfunction and loss of oBRB (Figures 2D and S4). Together, the data shows that loss of ZO-1 and ZO-2 in DKO mice retina affects the oBRB integrity.

### Loss of zonula occludens-1 and zonula occludens-2 results in the disruption of the retinal architecture as assessed by transmission electron microscopy

**Figure 3:**
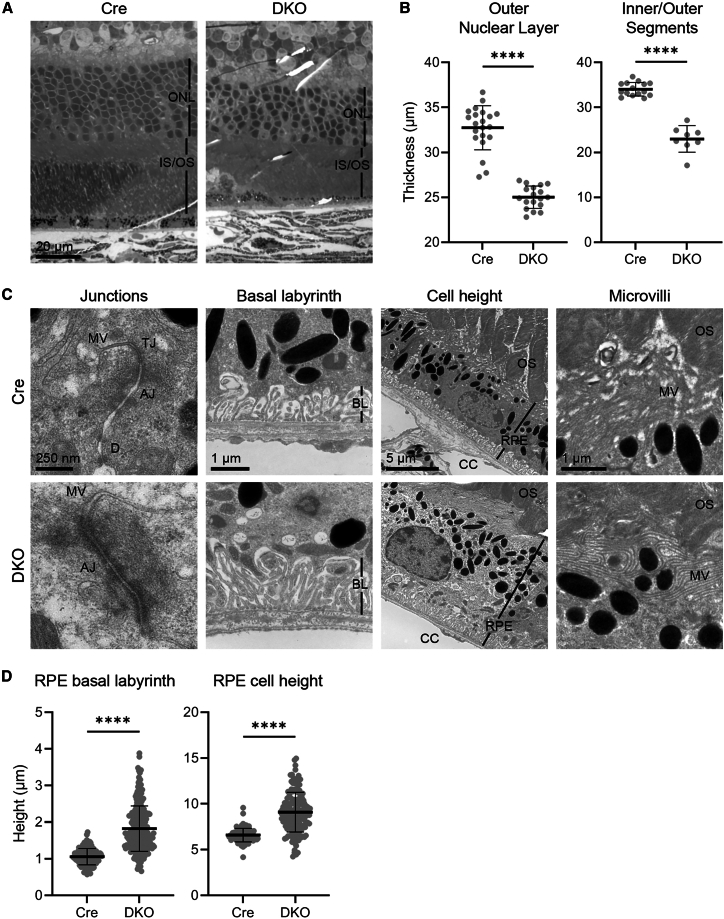
Changes in the retina and RPE of DKO mice 1-month post-Dox using transmission electron microscopy (TEM) (A) Thinning of the photoreceptor layer in DKO mice (right) compared to Cre control (left). ONL = Outer Nuclear Layer. IS/OS = Photoreceptor Inner/Outer Segments. (B) Quantification of the thickness of ONL (outer nuclear layer) and inner/outer segments is shown in (A). (C) Morphology of the RPE in Cre control (top row) and DKO (bottom row). First column: tight junctions (TJ) are absent in DKO; AJ = adherens junctions, D = desmosomes, MV = microvilli. In the second and third columns: the basal labyrinth (BL) and RPE cell height are increased in DKO. Last column: microvilli (MV) appear less compact in DKO. OS = Photoreceptor Outer Segments. CC = Choriocapillaris. (D) Quantification of the height of the RPE basal labyrinth (left) and RPE cell layer (right) shown in (C). Data for (B) and (D) are represented by mean ± SEM. Statistical analyses for (B) and (D) were performed using a two-tailed Student’s unpaired *t* test. (∗∗∗∗) = *p* < 0.0001.

To examine the ultrastructural changes arising from the loss of ZO-1/2 proteins, we performed transmission electron microscopy (TEM) on DKO mice retina at 1-month post-Dox. TEM images of DKO mice retina revealed significant thinning of the outer nuclear layer (ONL) (Cre control: 32.7 ± 2.4 μm vs. DKO: 25.0 ± 1.2 μm vs., *p* < 0.0001), as well as the photoreceptor inner/outer segments (IS/OS) (Cre control: 34.1 ± 1.5 μm vs. DKO: 23.0 ± 2.9 μm, *p* < 0.0001), when compared to the Cre control mice (Figures 3A and 3B). This suggests that the loss of ZO-1/2 proteins in the DKO RPE leads to photoreceptor atrophy. On the cellular level, we confirmed the absence of TJs in DKO mice RPE. In TEM, TJs in healthy RPE appear as electron-dense membrane contacts at the apical most tip of the lateral membranes.^26^ These TJs are followed basally by AJs, which can be discriminated from TJs by the increased width of the paracellular space and an electron-dense cytoplasmic plaque composed of actin networks. In Cre control RPE, TJs and AJs were clearly evident (Figures 3C and S5A). In contrast, TJs were absent in DKO RPE. Instead, AJs appeared to be extended and were located directly at the border between the lateral membrane and the microvilli-rich apical domain. This suggests a complete loss of the TJ structure in the DKO RPE. Furthermore, in DKO mice we observed: (i) an increase in the height of the basal labyrinth (1.8 ± 0.6 μm vs. Cre control 1.1 ± 0.2 μm, *p* < 0.0001); (ii) an increase in RPE cell height (9.1 ± 2.2 μm vs. Cre control 6.6 ± 0.7 μm, *p* < 0.0001) (Figures 3D, S5B, and S5C); (iii) a reduction in the density of apical microvilli (Figures 3C and S5D). Together, these results confirm that the loss of ZO-1 and ZO-2 disrupts the TJs in the RPE, resulting in structural anomalies, oBRB dysfunction, and photoreceptor layer atrophy.

### Loss of zonula occludens-1 and zonula occludens-2 disrupts retinal pigment epithelium molecular phenotype and enhances cell proliferation

**Figure 4:**
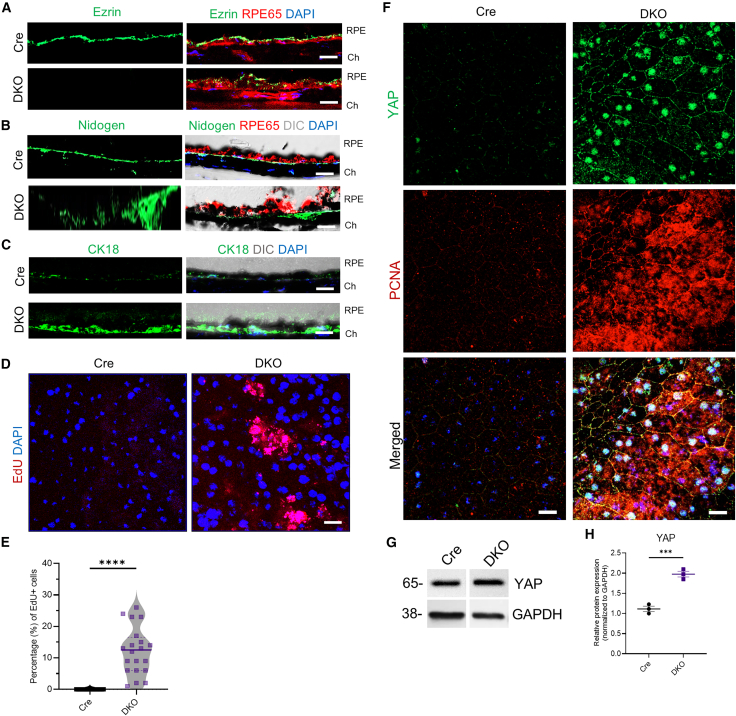
Molecular changes in DKO mice RPE at 1-month post-Dox (A) IF staining of Ezrin (green) indicated a loss of apical localization in RPE (marked by RPE65, red) in DKO mice. Ch = Choroid. (B) IF staining of Nidogen (green) indicated a loss of basal localization in RPE (marked by RPE65, red) in DKO mice. (C) IF staining of Cytokeratin 18 (CK18, green) showed an upregulation in DKO mice RPE (indicated by the top pigmented layer, DIC). (D) Increased EdU incorporation in DKO RPE cells. (E) Quantification of EdU positive nuclei in the RPE was performed and showed an increased percentage (%) of proliferating RPE cells in DKO mice (*n* = 20 images per genotype). (F) IF staining of YAP (green) and PCNA (red) showed increased co-expression in DKO RPE. (G) Western blot analysis showed an increased expression of YAP and PCNA in the DKO RPE lysates. (H) The relative increase in the protein expression of YAP and PCNA in DKO RPE (*n* = 3 eyes) was quantified. Data for (E) and (H) are represented by mean ± SEM. Scale bar for (A – C), 10 μm; for (D), 20 μm; for (F), 40 μm. Statistical analyses for (E and H) were performed using a two-tailed Student’s unpaired *t* test. (∗∗∗) = *p* < 0.001, (∗∗∗∗) = *p* < 0.0001.

Due to the significant cellular and tissue-level changes observed by TEM, we next analyzed the RPE molecular phenotype by the co-labeling of RPE65 (a mature RPE visual cycle protein)^27^ with either Ezrin (an apical microvilli marker)^28^ or Nidogen (a basal membrane marker).^29^ In line with the ultrastructural findings, both distinctly localized markers in control RPE appeared fragmented and discontinuous in RPE lacking ZO-1/2 (Figures 4A and 4B). This suggests the loss of the RPE phenotype and potentially cellular orientation. Cellular mis-orientation has been reported to affect the RPE post-mitotic status, trigger re-entry into the cell cycle, and induce proliferation.^30^ To investigate if this was also the case in RPE lacking ZO-1/2, we studied the expression of cytokeratin 18 (a marker of proliferation)^31^ and analyzed 5-ethynyl-2′-deoxyuridine (EdU) incorporation in DKO mice. Consistent with our hypothesis, we detected the increased expression of cytokeratin 18 (Figure 4C) and EdU-positive nuclei (12.20 ± 1.7%, *p* < 0.0001) in RPE flatmounts of DKO mice (Figures 4D and 4E). In addition, in DKO RPE, we detected the enhanced nuclear localization of YAP, a known regulator of cell proliferation,^32^ in proliferating RPE cells that co-labelled with PCNA (Figure 4F). Upon quantifying the protein levels of YAP from RPE lysates using Western blot analysis, we observed a 1.97-fold increase (*p* < 0.001) in expression in DKO mice RPE compared to Cre control (Figures 4G and 4H). Together, the results indicate that the absence of the TJ complex proteins ZO-1 and ZO-2 switches RPE’s post-mitotic phenotype to a proliferative phenotype.

### Progressive photoreceptor degeneration with age in double knockout mice

**Figure 5:**
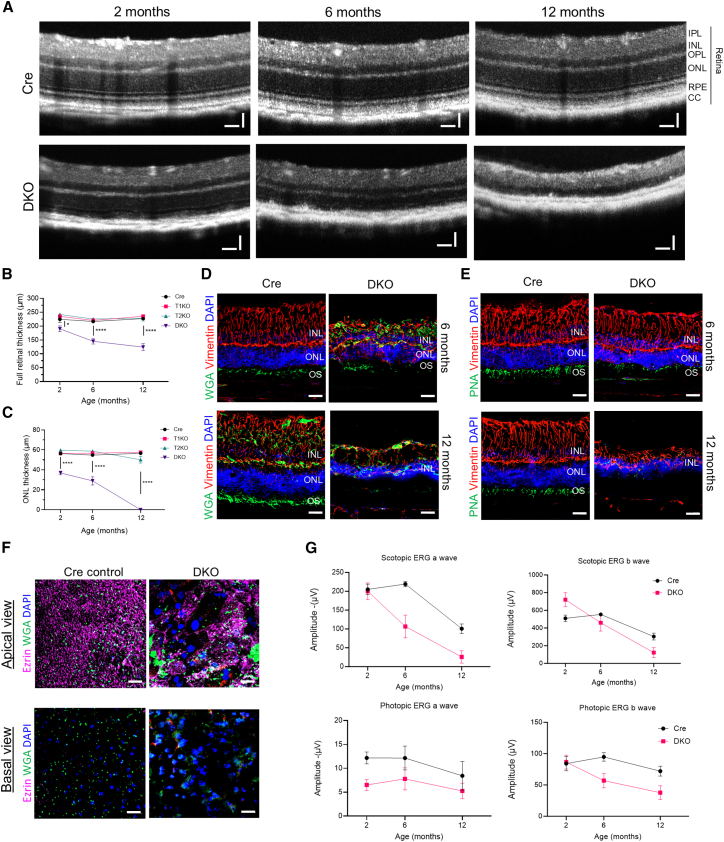
Progressive photoreceptor degeneration in 1-year-old DKO mice (A) Optical coherence tomography (OCT) of Cre and DKO mice at 2, 6, and 12 months (Dox administered on 1-month-old mice). DKO showed the progressive thinning of the retina for up to 12 months. (B and C) Automated layer segmentation of 6–18 eyes per genotype was performed to quantify the (B) full retinal and (C) ONL thickness, indicating a reduction in both for DKO with time. (D) IF of rod POS (WGA, green), and Müller glia (Vimentin, red) showed loss and disorganization in DKO mice at 6 and 12 months. (E) IF of cone POS (PNA, green), and Müller glia (Vimentin, red) showed a loss and disorganization in DKO mice at 6 and 12 months. (F) IF of RPE flat mount using WGA (green) to mark rod photoreceptor OS and Ezrin (purple) to mark the apical surface. Both the “apical view” and the “basal view” are shown. WGA puncta are observed in both views only for Cre mice, but not DKO. (G) Quantification of scotopic a- and b-waves (top row) and photopic a- and b-waves (bottom row) at 2, 6, and 12 months in Cre and DKO mice (*n* = 12 eyes per genotype). Data for (B), (C), and (G) are represented by mean ± SEM. Scale bars for (A), 50 μm; for (D, E), 40 μm; for (F), 10 μm. Statistical analyses for (B and C) were performed using a two-tailed Student’s unpaired *t* test. (∗) = *p* < 0.05, (∗∗∗∗) = *p* < 0.0001. IPL = Inner Plexiform Layer. INL = Inner Nuclear Layer. OPL = Outer Plexiform Layer. ONL = Outer Nuclear Layer. RPE = Retinal Pigment Epithelium. CC = Choriocapillaris. OS = Outer Segments.

We postulate that the ultrastructural changes in apical microvilli of RPE and the oBRB dysfunction observed may progress to outer retinal degeneration with age in the DKO mice. Indeed, optical coherence tomography (OCT) images revealed a progressive reduction in thickness from 2 to 12 months (Dox treatment given on 1-month-old mice) across all retinal layers in DKO mice (12 months, DKO: 124 ± 47 μm vs. Cre: 226 ± 11 μm), with complete loss of the outer nuclear layer in DKO retina by 12 months (Figures 5A–5C). Whereas, in T1KO and T2KO, the retinal thickness remained comparable to the Cre control (Figure S6), suggesting that loss of both ZO-1/2 was required for the retinal degeneration. The retina of a 12-month-old DKO mice was further visualized using H&E, where we observed RPE cell loss and photoreceptor degeneration (Figure S7). Whilst, as expected, Cre control and the sKOs (T1KO and T2KO) retina remained structurally normal. To confirm the extent of the loss of the ONL (i.e., photoreceptor nuclei) in DKO mice, we performed immunostaining of 6-month- and 12-month-old mice using wheat agglutinin (WGA), a marker of rod photoreceptor outer segments (POS), and peanut agglutinin (PNA), a marker of cone POS.^33^ Staining for both markers gradually decreased between 6 and 12 months post-Dox treatment in DKO retinas, consistent with a gradual loss of cone and rod POS (Figures 5D and 5E). Likewise, the expression of vimentin, a Müller glia marker,^34^ was reduced with time, further suggesting broader retinal atrophy. PR degeneration may be a consequence of dysfunctional RPE cells lacking ZO-1/2 to phagocytose cone and rod PRs. Indeed, reduced the expression of Ezrin (marker of apical microvilli) and WGA, both apically and internally in DKO RPE cells, suggests compromised phagocytic activity (Figure 5F). Full field electroretinogram (ERG) was performed to corroborate the morphological changes affecting global retinal function in DKO mice. In dark-adapted scotopic conditions, the ERG amplitude of a-wave (mixed response from rod and cone cells) progressively decreased from 106 μV to 25 μV, compared to 219 μV and 100 μV in Cre control mice at 6 and 12-month, respectively (Figures 5G and S8). The scotopic b-wave amplitude (generated from bipolar and muller cells) was also reduced from 553 μV to 303 μV in Cre mice, and from 459 μV to 122 μV in DKO mice, at 6 and 12 months, respectively. In photopic condition, the ERG amplitude of a-wave (Cre: 12.2 μV and 8.4 μV; DKO: 7.8 μV and 5.3 μV) and b-wave (Cre: 94 μV and 72 μV; DKO: 57 μV and 37 μV) was also reduced in DKOs at both 6 and 12 months (Figure S9).

### Aberrant retinal pigment epithelium proliferation and delayed wound repair in a laser-induced injury model

**Figure 6:**
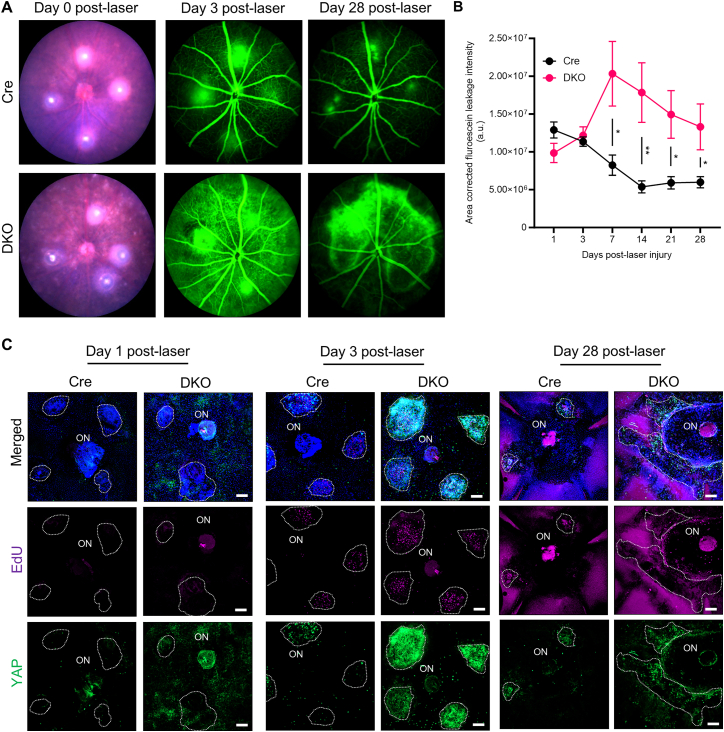
Hyper-proliferative RPE delays wound healing in the laser-induced CNV model (A) Laser-induced CNV in Cre control and DKO mice (1-month post-Dox). Fundus photographs were taken at day 0 post-laser and show the four laser spots. Fundus fluorescein angiography (FFA) at day 3 and day 28 post-laser shows leakage from the lasered spots. (B) Area corrected net fluorescein leakage area (μm) from the lasered spots (*n=*5 to 8 eyes per genotype) was quantified at regular intervals (days 1, 3, 7, 14, 21, and 28 post-laser) and displayed increased leakage with time for DKO, in contrast to Cre control. Data are represented by mean ± SEM. (C) YAP expression, and proliferation status (monitored by EdU) of RPE surrounding the lasered spots at days 1, 3, and 28 post-laser. There was increased EdU incorporation in DKO mice at day 3 post-laser. By day 28, the observed region of EdU-positive cells is much larger than in Cre mice, indicating aberrant hyper-proliferation. Dashed white lines indicate the region surrounding the laser spots. ON: Optic nerve. Scale bar, 100 μm. Statistical analysis for (B) was performed using a two-tailed Student’s unpaired *t* test. (∗) = *p* < 0.05, (∗∗) = *p* < 0.01.

The aberrant proliferation of post-mitotic RPE has been observed in retinal pathogenesis including AMD, PVR, and diabetic retinopathy (DR).^35^^,^^36^ In mice, laser-induced damage activates RPE proliferation, resulting in spontaneous wound healing.^37^ Since the loss of ZO-1 and ZO-2 activates RPE cell proliferation (Figures 4D–4G), we explored if this affects normal wound healing in response to laser-induced photocoagulation and choroidal neovascularization (CNV).^37^^,^^38^^,^^39^ For this purpose, Cre control and DKO mice eyes (*n* = 16 eyes per genotype) were injured with four laser spots. CNV formation and vascular leakage were monitored using live ophthalmic imaging for 28 days after laser treatment. In Cre control mice, quantitative analysis of fluorescein leakage indicates maximal leakage at day 1, with progressive resolution from day 3 to day 14, with near complete resolution by day 28 (Figures 6A and 6B). In contrast, in DKO eyes, vessel leakage gradually increased beyond day 1 and peaked at day 7, with persistent leakage until day 14–28. The resolution of laser induced CNV lesions in mice has been reported to occur due to robust RPE proliferation.^40^ Consistent with this, we observed increased area of EdU incorporation in the Cre control mice at day 3 post-laser injury that subsided by day 28 (Figure 6C). Conversely, the area with sustained EdU incorporation adjacent to the laser spot was noted in DKO RPE beyond day 3 and up to day 28. YAP has been reported to play a role in CNV formation through the regulation of endothelial cells,^41^ however its role in RPE in CNV is unknown. We observed that YAP expression levels and nuclear localization were increased in DKO retinas post-CNV induction and remained elevated till day 28. Taken together, these results indicate that wound-healing in the laser-induced CNV model is deregulated in retinas lacking ZO-1/2. This may possibly be due to the dysregulation of YAP, amongst other factors, resulting in aberrant wound healing and scarring.

## Discussion

TJ complexes are thought to play a pivotal role in retinal homeostasis by upholding the integrity of the oBRB, which comprises the RPE, Bruch’s membrane (BM) and choroid.^42^ Although the dysfunction of oBRB has been associated with many retinal diseases,^42^ it is less clear if oBRB dysfunction is a cause or consequence of disease pathogenesis. In particular, the role of each specific TJ component in the RPE remains elusive. In this study, we generated inducible, RPE-specific ZO-1/Tjp1 (T1KO), ZO-2/Tjp2 (T2KO) and DKO knockout mouse models. While the inactivation of ZO-1 or ZO-2 alone had no major overt phenotype, DKO exhibited the following profound phenotype including: (i) a compromised oBRB structure and function; (ii) alterations of the apical domain, cellular orientation, and cell morphometry of RPE; (iii) progressive photoreceptor degeneration; and (iv) impaired wound healing in the laser-induced CNV model. The oBRB was disrupted in DKO mice, as evidenced by the hyper-fluorescence of FFA *in vivo* and leakage of FITC-dextran into the retina through RPE monolayer discontinuities seen in histology. By TEM, characteristic electron-dense TJ plaques were absent in DKO retina, compared to control RPE. Our findings are consistent with previous observations, whereby inactivation or silencing of both ZO-1 and ZO-2 are required to disrupt the paracellular barrier function, both *in vitro* in MDCK/EPH4 epithelial cell culture and *in vivo* in liver tissue.^13^^,^^43^^,^^44^ In addition, our study also suggests the redundancy of ZO-1/2 in the retina.

ZO proteins, as cytoplasmic adaptors, link transmembrane TJ proteins to the cytoskeleton to maintain barrier function. Despite their structural similarity, various studies have reported non-redundant functions of ZO-1 and ZO-2 proteins in different tissue contexts. This can include their tissue distribution^45^^,^^46^ to role in paracellular permeability,^47^ and even embryonic lethality when individual knockouts are performed.^10^ However, in our study, T1KO and T2KO did not differ significantly in terms of RPE cellular morphometry, overall RPE and retinal structure for up to 12 months, suggesting a high level of functional redundancy between ZO-1 and ZO-2. While we cannot rule out subtle ultrastructural alterations in T1KO and T2KO, the absence of overt structural or functional defects would make it difficult to attribute ultrastructural differences, if any, to biologically meaningful effects. On the other hand, in DKO mice, we observed that the loss of ZO-1/2 and oBRB, was sufficient to lead to profound retinal degeneration. We demonstrated that this is likely due to the loss of epithelial cell orientation, changes in basal labyrinth structure, and reduced apical microvilli density that occurred 1-month post-Dox. Tight junctions establish physiological barriers that regulate the movement of small solutes, ions, and fluid between tissue compartments. Therefore, the loss of ZO-1/2 leads to the dysregulation of the water transport and loss of ion gradients, which consequently causes loss of osmotic function of RPE cells as evidenced by increased cell and basal labyrinth height. Furthermore, the localization of Ezrin – an apical marker that plays a key role in phagocytosis^48^ was disrupted in DKO RPE. Hence, loss of ZO-1/2 may also impair phagocytosis. The apical domain of the RPE is in intimate contact with the PR layer and plays a critical role in maintaining photoreceptor viability and function.^3^ The close association between RPE and PR cells is essential for the phagocytosis of distal photoreceptor outer segments (POS), which, if defective, leads to retinal degeneration. This is observed in dry AMD, whereby RPE dysfunction leads to photoreceptor loss and ultimately vision impairment.^49^ In our study, we observed age-dependent progressive thinning of the retina, in particular, the loss of ONL in the DKO. This is possibly due to defects in the RPE-PR interaction and POS phagocytosis, thus supporting the critical role of an intact RPE for retinal health. Full-field ERG confirmed that these morphological changes impair global retinal function. This suggests that DKO mice may provide a model for the progressive degeneration of the photoreceptors induced by a defective RPE, a hallmark of retinal degenerative diseases.^50^^,^^51^

We observed that the inactivation of ZO-1 and ZO-2 re-activated RPE cell proliferation. This was accompanied by increased protein levels and nuclear localization of YAP – a known transcriptional regulator of cell proliferation and tissue size.^52^^,^^53^ This is unexpected, as under normal physiological conditions, polarized RPE is post-mitotic and does not proliferate due to contact inhibition.^36^^,^^54^ ZO-1 and ZO-2 have been shown to interact with YAP. In particular, ZO-2 plays a role in the cytoplasmic retention of YAP, thereby suppressing YAP’s nuclear translocation and transcriptional activity.^55^^,^^56^^,^^57^ However, its role in RPE proliferation and wound healing has not been previously described. We demonstrated that ZO proteins may be implicated in wound healing via the regulation of YAP. In the widely used laser-induced CNV model, the mouse retina has been described to heal spontaneously.^39^ We observed that this healing process was characterized by a short transient upregulation of YAP expression, with cell proliferation in the normal retina. This is in contrast with DKO retina, whereby YAP expression levels persisted in the injured DKO retina, which could have contributed to cell dysmorphia, persistent cell proliferation, and tissue scarring. This suggests that in the absence of ZO-1/2, the loss of contact inhibition after wound healing may induce the persistent activation of YAP and trigger RPE cells to undergo EMT, to promote scarring. However, the presence of other co-drivers aside from YAP cannot be precluded and warrants further investigation to elucidate the complete mechanism. Nevertheless, previous studies have implicated ZO proteins in oral mucosa and skin repair upon injury.^58^ Therefore, this DKO model also presents utility in screening for anti-scarring targets, with potential therapeutic application for end-stage AMD, whereby scarring is a common complication.

In conclusion, the RPE-specific inactivation of both ZO-1/Tjp1 and ZO-2/Tjp2 results in the loss of the structural and functional integrity of the oBRB and RPE cell architecture, with progressive effects on photoreceptor health and vision. Upon injury, the ZO-1/2 deficient RPE sustains YAP upregulation and cell proliferation and fails to re-establish contact inhibition after healing. The DKO mice may provide a disease model for progressive retinal degeneration and sub-retinal fibrosis, secondary to RPE dysfunction, such as end-stage AMD and central serous chorioretinopathy (CSR).

### Limitations of the study

One limitation of our study is that although we observed sustained co-localization of YAP with EdU-positive RPE cells in the DKO model, the precise molecular mechanisms by which YAP contributes to RPE hyper-proliferation and delayed wound healing remain undefined. Future studies will focus on delineating the upstream regulators and downstream effectors of YAP signaling in this context.

## Resource availability

### Lead contact

Further information and requests for the resources and reagents should be directed to and will be fulfilled by the lead contact, Xinyi Su (su_xinyi@a-star.edu.s).

### Materials availability

This study did not generate new unique reagents.

### Data and code availability

• This article does not report original code.
• Any additional information required to re-analyze the data reported in this article is available from the lead contact upon request.
• All other items: No other items were generated in this study.

## Acknowledgments

The authors would like to thank the A∗STAR Microscopy Platform (AMP) for providing microscope resources and the Advanced Molecular Pathology Laboratory (AMPL) at the Institute of Molecular and Cell Biology (IMCB), A∗STAR, for kindly providing support with histological processing. TEM sectioning and imaging were performed at the NTU Institute of Structural Biology (NISB) Cryo-EM lab at 10.13039/501100001475Nanyang Technological University, Singapore. We also acknowledge the support of the Mechanobiology in Epithelial 3D Tissue constructs (ME3T) graduate school (GRK 2415/363055819) for RPE cell morphometric analyses.

The work was supported by the Central Research Fund, Use-Inspired Basic Research by the Agency for Science, Technology and Research Biomedical Research Council (10.13039/501100001348A∗STAR, BMRC) [SC15/22-118400-0SXY] (XS), the Competitive Research Program (CRP) by 10.13039/501100001381National Research Foundation (NRF) [NRF-CRP21-2018-0008] (XS); GAP Funding by Agency for Science, Technology and Research Innovation & Enterprise (10.13039/501100001348A∗STAR I&E) [I23D1AG126] (XS), the 10.13039/501100001349National Medical Research Council (NMRC) Open Fund – Large Collaborative Grant by the 10.13039/501100001350Singapore Ministry of Health [OFLCG23may-0032] (XS), the 10.13039/501100001459Ministry of Education (MOE) Academic Research Fund Tier 3 by MOE [MOE-T3-2020-001] (AL, XS), the 10.13039/501100001659German Research Foundation Grant – DFG (RU 337 2366/3-1) by the Interdisciplinary Centre for Clinical Research within the Faculty of Medicine at the 10.13039/501100007210RWTH Aachen University (JDR).

## Author contributions

Conceptualization: BHP, ZL, WH, and XS; funding acquisition: AL, WH, and XS; investigation: SMA, BHP, QSWT, BH, ANK, AB, JZ, DG, HB, SWY, DSLW, JX, KCT, ZL, VAB, KHC, and JDR; methodology: SMA, BHP, QSWT, BH, ANK, HB, DSLW, JX, YZL, JDR, WH, and XS; project administration: BHP; supervision: VAB, KHC, AL, WH, and XS; visualization: SMA, BHP, and QSWT; writing – original draft preparation: BHP, AB, BH, DG, AL, WH, and XS; writing – review and editing: BHP, BH, AB, KHC, ZL, AL, WH, and XS.

## Declaration of interests

The authors declare no competing interests.

## STAR★Methods

### Key resources table

**Table:** 

REAGENT or RESOURCE	SOURCE	IDENTIFIER
**Antibodies**		

Mouse anti E-Cadherin	BD Trans Labs	Cat# 610181; RRID: AB_397580
Mouse anti-GAPDH	EMD Millipore	Cat# MAB374; RRID: AB_2107445
Mouse anti N-Cadherin	BD Trans Labs	Cat# 610920; RRID: AB_2077527
Mouse anti-RPE65	Abcam	Cat# ab13826; RRID: AB_ 2181006
Rabbit anti-active Yap1 [EPR19812]	Abcam	Cat# ab205270; RRID: AB_2813833
Rabbit anti-Claudin-2	Life Technologies	Cat# 51-6100; RRID: AB_2533911
Rabbit anti-Collagen1A1	Cell Signalling Tech	Cat# 72026; RRID: AB_2904565
Rabbit anti-Cytokeratin 18	Abcam	Cat# ab32118; RRID: AB_736394
Rabbit anti-Ezrin	Abcam	Cat# ab41672; RRID: AB_941504
Rabbit anti-Laminin	Abcam	Cat# ab11575; RRID: AB_298179
Rabbit anti-OTX2 [EPR3348]	Abcam	Cat# ab92326; RRID: AB_10562130
Rabbit anti-Vimentin	Abcam	Cat# ab15248; RRID: AB_301789
Rabbit anti-ZO1	Life Technologies	Cat# 61-7300; RRID: AB_2533938
Rabbit anti-ZO2 (C-terminus)	Life Technologies	Cat# 38-9100; RRID: AB_2533390
Rat anti-Nidogen	Millipore	Cat# MAB1946; RRID: AB_94438
Rat anti-ZO1	DSHB	Cat# R26.4C; RRID: AB_2205518

**Chemicals, peptides, and recombinant proteins**		

2.5% glutaraldehyde	Agar Scientific	Cat# AGR1010
Acetone	Fisher Chemical	Cat# A18-1
Alexa Fluor 568/647 Phalloidin	Thermo Fisher Scientific	Cat# A12380
Alexa Fluor 647 Phalloidin	Thermo Fisher Scientific	Cat# A30107
Araldite resin	Electron Microscopy Sciences	Cat# 10900
BSA	Sigma-Aldrich	Cat# MFCD00130384
DAPI	Thermo Fisher Scientific	Cat# D1306
Doxycycline Hyclate	Sigma-Aldrich	Cat# D9891
Fluorescein Isothiocyanate–Dextran ((average mol wt 40,000)	Sigma	Cat# FD40
Hoechst 33342	Thermo Fisher Scientific	Cat# 62249
Osmium Tetroxide	Ted Pella	Cat# 18463
Paraformaldehyde	Sigma-Aldrich	Cat# 158127
Phenylephrine	Bausch and Lomb	Cat# 9793400
ProLong Gold Antifade	Thermo Fisher Scientific	Cat# P36934
Reynold’s lead citrate (prepared with lead nitrate and sodium citrate)	Electron Microscopy Sciences	Cat# 22410
Sodium fluorescein	SERB Pharmaceuticals	Cat# SIN13314P
TritonX-100	Promega	Cat# H5141
Tropicamide	Alcon	Cat# NDC 0998-0355-15
Tween-20	Promega	Cat# H5151
Uranyl Acetate	Electron Microscopy Sciences	Cat# 22400
Uranyl Acetate	Electron Microscopy Sciences	Cat# 22400

**Critical commercial assays**		

Click-iT EDU kit	Life Technologies	Cat# C10634
iScript Reverse Transcription Kit	Bio-Rad	Cat# 1708841
KAPA SYBR FAST qPCR Master Mix (2X) Kit	Sigma-Aldrich	Cat# KK4600

**Experimental models: Organisms/strains**		

Mouse: *VMD2*-Cre (‘RPE-Cre’) in C57Bl6/J background strain	University of Oklahoma Health Sciences Center	Nil
Mouse: *Tjp1*^f/f^·RPE-Cre^+^ (T1KO); *Tjp2*^f/f^·RPE-Cre^+^ (T2KO); and *Tjp1*^f/f^/*Tjp2*^f/f^·RPE-Cre^+^ (DKO)	Agency for Science, Technology, and Research Biological Resource Centre	Nil

**Oligonucleotides**		

*Tjp1* forward primer: 5’ AGCGAATGTCTAAACCTGGG 3’	Integrated DNA Technologies	Nil
Tjp1 reverse primer: 5’ TCCAACTTGAGCATACACAGG 3’	Integrated DNA Technologies	Nil
*Tjp2* forward primer: 5’ TCAACATCCCAGCCCTAAAC 3’	Integrated DNA Technologies	Nil
*Tjp2* reverse primer: 5’ CTCTCCTTCAGCTTCTCAGTG 3’	Integrated DNA Technologies	Nil
*ACTB* forward primer: 5’ ACCTTCTACAATGAGCTGCG 3’	Integrated DNA Technologies	Nil
*ACTB* reverse primer: 5’ CTGGATGGCTACGTACATGG 3’.	Integrated DNA Technologies	Nil

**Software and algorithms**		

Axio Imager.A2	Zeiss	Nil
Cellpose	Nil	https://www.cellpose.org/
Espion system	Diagnosys LLC	Nil
Fiji software	Nil	https://imagej.net/software/fiji
GraphPad Prism (v10.4.2)	Nil	https://www.graphpad.com/
Micron IV Rodent Comprehensive imaging system	Phoenix Micron, USA	Nil
Tecnai T12	FEI / Thermo Fisher Scientific	Nil
Zen 3.4	Zeiss	Nil

**Other**		

A2 microscope equipped with a digital camera	Zeiss	Nil
Espion system	Diagnosys LLC	Nil
Formvar	Electron Microscopy Sciences	Nil
LSM 800 confocal microscope	ZEISS	Nil
Tecnai T12 imager with CCD camera	FEI / Thermo Fisher Scientific/ Olympus Soft Imaging Solutions	Nil
Ultramicrotome	Leica Microsystems	Nil
Veleta CCD camera	Olympus Soft Imaging Solutions	Nil

### Experimental model and study participant details

All animal experiments in this study comply with the ARRIVE guidelines. All protocols that are related to any mice work are in accordance with the Institutional Animal Care and Use Committee (IACUC) protocol #201558 approved by the IACUC committee of Agency for Science, Technology and Research Biological Resource Centre (A∗STAR BRC). All mice used were from C57Bl6/J background strain. Mice were housed under a 12-h/12-h light/dark cycle and raised in groups of up to five mice each. The RPE-Cre^+^ mice were crossed over with the *Tjp1*^flox/flox (f/f)^ and *Tjp2*^f/f^ mice^17^ to obtain the following mice strain: *Tjp1*^f/f^·RPE-Cre^+^ (T1KO), *Tjp2*^f/f^·RPE-Cre^+^ (T2KO) and *Tjp1* ^f/f^/*Tjp2*^f/f^·RPE-Cre^+^ (DKO). The Cre expression is driven by the human *VMD2* promoter-controlled rtTA. *VMD2* promoter directs the RPE-specific expression of the tet system transactivator gene rtTA. The rtTA is designed to activate tetO-controlled Cre expression in the presence of doxycycline (Dox) in the RPE, thereby generating cell-specific KO. Male and female mice of equal numbers were used for the experiments described unless specifically stated otherwise. All mice were induced at 1-month post-natal with 1.5 mg/mL of doxycycline (Dox) injected intra-peritoneally for 5 consecutive days and eyes were harvested after 4 weeks to check for deletion of ZO-1/*Tjp1* and ZO-2/*Tjp2* using western blot, RT-qPCR and IF.

### Method details

#### Western blot of RPE-choroid tissue

Enucleated mice eyes were immediately dissected to remove the anterior segment containing the cornea, iris, and lens. After removing the neural retina, the eye cup with RPE still attached to the choroid and sclera were inverted and transferred to a separate tube with RIPA lysis buffer and placed on ice. The tube containing the eye cup was tapped intermittently for a period of 10 min to release the RPE. To complete the lysis, the tube was vortexed vigorously before centrifuging to obtain the supernatant. After centrifuging at 10,000 rpm for 10 min, the supernatant was transferred to a new tube. The protein sample was quantified using Bradford assay. RPE lysate from each eye was sufficient for one run in a single lane. The antibodies used to detect the various proteins include rabbit ZO-1 (Life Technologies), rabbit ZO-2 (Life Technologies), mouse RPE65 (Abcam), and mouse GAPDH (Millipore) (Table S1). For each of the 4 genotypes, three biological replicates (i.e. 3 mice) were selected. All expression levels were normalized to those of *Gapdh* as the loading control.

#### Reverse transcription quantitative polymerase chain reaction (RT-qPCR)

Enucleated mice eyes were immediately dissected to remove the anterior segment containing the cornea, iris, lens, and neural retina. To extract RNA, both eyecups from the same mouse were transferred into a microcentrifuge tube containing 350 μL of Buffer RLT (Qiagen) supplemented with 1% β-mercaptoethanol (Sigma-Aldrich) and incubated for 10 min at room temperature. The lysate mixture was gently agitated by tapping the tube against the benchtop to dislodge the pigmented RPE cells during the incubation period, after which, the eyecups were removed from the mixture.^59^ The lysate was vortexed for 1 min to homogenize, then centrifuged at 16000 g for 4 min at 4 °C. The supernatant was then transferred into a fresh microcentrifuge tube and purified for total RNA using RNeasy Micro Kit (Qiagen) as per the manufacturer’s recommended protocol. 150 ng of purified RNA was reverse transcribed into cDNA using the iScript Reverse Transcription Kit (Bio-Rad), followed by quantitative PCR (qPCR) with gene-specific primers and KAPA SYBR FAST qPCR Master Mix (2X) Kit (Sigma-Aldrich) on a QuantStudio 5 Real-Time PCR System (ThermoFisher Scientific). For each of the 4 genotypes, three biological replicates were selected, and each replicate was analysed in technical triplicates. The comparative Ct method was employed in analysing the relative genetic expression. All expression levels were normalized to those of *Actb* (β-Actin) gene. Forward (F) and reverse (R) Primers used, *Tjp1*: F – 5’ AGCGAATGTCTAAACCTGGG 3’ and R – 5’ TCCAACTTGAGCATACACAGG 3’; *Tjp2*: F – 5’ TCAACATCCCAGCCCTAAAC 3’ and R – 5’ CTCTCCTTCAGCTTCTCAGTG 3’; *ActB*: F – 5’ ACCTTCTACAATGAGCTGCG 3’ and R – 5’ CTGGATGGCTACGTACATGG 3’.

#### Immunofluorescence (IF) of RPE-choroid flat mounts

The mouse eyeballs were enucleated and fixed in 4% paraformaldehyde (PFA, Sigma Aldrich) in PBS for 2 min. Once fixed, the eyeballs were punctured at the limbal region before removing the cornea and lens. The neural retina was separated from the RPE-choroid eyecup for staining. RPE-choroid tissue was cut into 4 petals and placed in methanol until immunostaining. The RPE-choroid tissue was removed from the methanol and washed in PBS before incubating with a blocking solution composed of 0.1% BSA (Sigma-Aldrich) in 3% TritonX-100 and 1% Tween-20 in PBS. After 1 h of blocking, the flat mounts were incubated with primary antibodies (Table S1) in the same blocking solution and incubated overnight at 4 °C. The flat mounts were then washed in blocking solution 6 times with an interval of 10 minutes. Respective Alexa Fluor secondary antibodies were added in the same blocking solution and left at room temperature for 1 h. After which, in some samples, Alexa Fluor 568/647 Phalloidin (Thermo Fisher Scientific) was added for 10 min before proceeding with washing 4 times in PBS with an interval of 10 min. A secondary antibody only negative control was included where sections were only incubated with a blocking buffer in place of primary antibodies. Nuclei were stained with Hoechst 33342 (Thermo Fisher Scientific) or DAPI (Thermo Fisher Scientific). The flatmounts were mounted using ProLong Gold Antifade (Thermo Fisher Scientific) and stored at 4 °C until imaging. Images were taken using LSM800 confocal laser microscope (Zeiss).

#### Segmentation and shape index quantification of RPE cells

Confocal microscopy images of RPE cells captured on RPE-choroid flat mounts were processed and analyzed following a standardized workflow. To enhance the signal from intercellular junctions, maximum intensity projects were created using Fiji software. Cell segmentation was performed using the deep-learning-based software Cellpose.^60^ The pre-trained “cyto” model was selected, optimized for cytoplasmic features, with the estimated cell diameter set to 60 pixels. The segmentation process generated binary masks representing individual cell outlines (marked with Phalloidin staining), which were exported as outlines in a text format compatible with Fiji. The exported outlines were imported into Fiji as Regions of Interests (ROIs) for further morphological analysis. Skeletonized representations of cells were generated to visualize cellular shapes and confirm segmentation quality. ROIs corresponding to cells at the edges of the images were excluded from analysis to avoid segmentation artefacts.

For each segmented cell, area (A) and perimeter (P) were measured using Fiji’s measurement tools. These values were used to calculate the cellular shape index (q), a dimensionless metric defined as:

$$q=\frac{P}{\sqrt{A}}$$

This index quantifies cell morphology, where higher values indicate more irregular or elongated cells. Statistical analysis was performed using GraphPhad Prism. Data was tested for normality before proceeding with further statistical analyses. Morphological differences between experimental groups and a control sample were assessed using Kruskal-Wallis test with Dunn’s multiple comparison test, with significance set at *p* < 0.05.

#### Live ophthalmic imaging

Mice that were 2-months and/or 12-month-old (Dox induction at 1-month-old) were anaesthetized with a combination of ketamine (150 mg/kg BW) and xylazine (10 mg/kg). The pupils were dilated with 1% tropicamide followed by 1% phenylephrine. Colored fundus photography (FP), fundus fluorescein angiography (FFA), and optical coherence tomography (OCT) were performed using a Micron IV retinal imaging system (Phoenix Micron). After dilation, both eyes of the mice were first covered with a layer of vidisic gel to aid in FP and OCT imaging, and also to prevent drying of the cornea during procedure. For FFA, mice were intraperitoneally injected with 10% sodium fluorescein at a dose of 0.01 mL per 5 – 6 g body weight. Images were then taken at 5 mins post-injection. (InSight, Phoenix Technology). The full retinal thickness (FR) and outer nuclear layer (ONL) were automatically segmented and then manually adjusted for accuracy. The thickness measurements were then exported onto a spreadsheet for further analysis.

#### Permeability assay using FITC-dextran

0.1 mL of 40 kD FITC-dextran (5 mg/mL) was injected on isoflurane-induced mice (1-month post-Dox, age: 2 months) using the retro-orbital injection method.^61^ 1 min post-injection, the mice were culled using the cervical dislocation and the eyes were enucleated and embedded for cryosectioning. The embedded eyes were sectioned using a cryotome with a thickness of 16 μm. Confocal microscopy was performed using LSM800 (Zeiss) to get a z-stack image for analysis of breakage points between the RPE and choroid. The images (*n*=5 per genotype) were quantified using ImageJ for fluorescence intensity measurement.

#### Hematoxylin and eosin (H&E) staining

Eyes from 2-months and 12-month-old mice were fixed in Davidson’s fluid for 16 h before paraffin embedding. 5 μm thick sections were cut using a microtome (Leica Microsystems) and stained with standard hematoxylin and eosin (H&E) protocol according to manufacturer’s instructions. Light micrographs were taken on Axio Imager.A2 microscope equipped with a digital camera (Zeiss).

#### Transmission electron microscopy (TEM)

##### Sample preparation

The enucleated mice eyes were fixed in an EM-grade fixative solution comprising 2% PFA (Sigma) and 2.5% glutaraldehyde (Agar Scientific) in 1X PBS overnight at 4 °C to preserve tissue integrity. The next day, retina samples were dissected and underwent an additional 2 h of fixation in the same freshly prepared fixative solution at 4 °C. Subsequently, the fixative solution was rinsed off with 1X PBS, and the samples were kept at 4 °C. The following day, the samples underwent EM processing and dehydration protocol at room temperature. This process began with osmium tetroxide fixation for 2 h, followed by sequential washes in deionized water and dehydration in increasing ethanol concentrations (25%, 50%, 75%, 95%, and 100%). The process concluded with 100% acetone treatment to ensure complete dehydration. Infiltration with Araldite resin (Electron Microscopy Sciences) was carried out at room temperature, gradually increasing the resin concentration mixed with acetone (acetone: resin in ratios of 1:1, 1:3, 1:6) overnight. The following day, transferred to 100% resin and proceeded with resin infiltration at various temperatures ranging from room temperature to 55 °C. Subsequently, embed the sample in a resin mold and polymerize it at 60 °C for a minimum of 24 h to ensure the sample achieves optimal hardness for sectioning.

##### Sectioning and staining

Sections with a thickness of about 75 nm were cut on an ultramicrotome (Leica Microsystems) using a diamond knife (Diatome) and picked up on 2x1 mm slot copper grids coated with formvar (Electron Microscopy Sciences). The samples were stained with Reynold’s lead citrate (LC) and uranyl acetate (UA) based on^62^ comprising the following steps: LC staining for 5 – 6 min; washing in boiled MilliQ water (boiled for ∼30 min to remove CO_2_; allowed to cool down completely before use), first on drops on Parafilm by dipping each grid 5x on three sequential drops, then in glass vials by dragging each grid 10x through the water surface of three sequential vials; air drying the samples completely; UA staining for 1 h; washing and drying as before; second LC staining for 20 min; washing and drying as before. LC was prepared with lead nitrate and sodium citrate (Electron Microscopy Sciences) according to^63^ using boiled MilliQ water; during the procedure 1 N NaOH (prepared using boiled MilliQ water) was added stepwise to ensure a final pH of 12. UA (Electron Microscopy Sciences) was prepared as 4% stock in water and centrifuged for 10 min at 15000 g before use. Staining was performed by floating the grids individually with the sections facing down on small drops of staining solution on Parafilm inside covered glass dishes. For LC staining sodium hydroxide pellets were added into the dishes to absorb CO_2_ and breathing onto the samples was strictly avoided to prevent the formation of precipitates.

##### Imaging

TEM imaging was carried out on a Tecnai T12 (FEI / Thermo Fisher Scientific) operated at 120 kV using a 2k x 2k Veleta CCD camera (Olympus Soft Imaging Solutions). Prior to recording micrographs, sections were irradiated at low magnification (690x) for a few minutes to prevent beam-induced shrinkage during imaging.

#### IF of retina cross-sections

Fresh/fixed-frozen eye sections are quenched in 50 mM ammonium chloride and permeabilized in 0.2% TritonX-100 in PBS before blocking in 1% BSA in 0.1% Tween20-PBS. Primary antibodies (Table S1) diluted in the blocking solution was added to the eye cup and incubated at 4 °C overnight and secondary antibodies diluted in the blocking solution were added to the eye cup and incubated at room temperature for 1 h. A secondary antibody only negative control was included where sections were only incubated with a blocking buffer in place of primary antibodies. Nuclei were stained with Hoechst 33342 (Thermo Fisher Scientific) or DAPI (Thermo Fisher Scientific). The flatmounts were mounted using ProLong Gold Antifade (Thermo Fisher Scientific) and stored at 4 °C until imaging. Images were taken using LSM800 confocal laser microscope (Zeiss).

#### EdU incorporation

Mice at 2 – 4 weeks post-Dox induction were injected intraperitoneally with daily doses of 200 μM of EdU for 3 days. The eyes were enucleated for analysis 24 h from the last EdU injection and staining using the click-iT EdU kit (Life Technologies) was done for the RPE flat mount. EdU that co-localized with nucleus was marked as an EdU positive cell.

#### Full-field electroretinography (ERG)

Standard full-field scotopic and photopic ERGs were recorded. Mice were dark-adapted for a minimum of 12 hours for scotopic recordings. All ERG recordings were performed in a dark room under dim red illumination using the Espion system (Diagnosys LLC). Mice were anesthetized and the eyes were dilated as described in “live ophthalmic imaging” methods. Their body temperature was maintained at 37 °C using a water heat pad fixed on the ERG stage. Coupling gel was applied to each eye before placing a monopolar electrode in the mouth to serve as the reference electrode, and a silver-silver chloride electrode was inserted into the tail as the ground electrode. The mouse was positioned in front of a Ganzfeld stimulus. Scotopic ERG responses were recorded at a light intensity of DA 10 cd.s.m^−2^. Photopic ERG responses were recorded at a light intensity of LA5 cd.s.m^-2^. Each response was an average of 6 trials. Signals were acquired at 2 kHz with high- and low-pass filtering at 0.3 and 100 Hz respectively.

#### Laser-induced choroidal neovascularization (CNV)

##### Laser induction

To induce CNV, 6- to 8-week-old mice were anesthetized with intraperitoneal injection of 50 mg/kg BW ketamine and 10 mg/kg BW xylazine. The pupils were dilated with 1% tropicamide followed by 2.5% phenylephrine. Laser induction was then done via the Micron IV Rodent Comprehensive imaging system (Phoenix Micron, USA) – 50 μm spot size, 0.05 seconds duration, 120 mW),^64^ 4 spots were given to each eye. FFA images were taken on day 3, 7, 14, 21 and 28 after laser treatment as described above.

##### Quantification of leakage

To quantify the CNV leakage, we utilized the capabilities of You Only Look at CoefficienTs (YOLACT), a state-of-the-art real-time instance segmentation model, to delineate regions of interest in retinal images.^65^ The training phase of this process involved an adjustment of the image dimensions to a uniform 544 x 544 pixels, followed by the min-max normalization. We adopted stochastic gradient descent with a momentum of 0.9 and an initial learning rate of 0.002, and three losses: classification loss (Lcls), box regression loss (Lbox),^66^ and mask loss (Lmask). The weights of Lcls, Lbox, and Lmask are 1, 1.5, and 6.125, respectively.

### Quantification and statistical analysis

GraphPad Prism software (version 10.4.2 for Windows, GraphPad Software, www.graphpad.com) was used to perform statistical analyses. Sample sizes for mice studies were determined mainly based on pilot studies. Results are reported as mean ± standard deviation (SD). For statistical analysis comparing two independent groups of data, two-tailed Student’s unpaired *t* test was used. For comparisons of data with more than two groups, a one-way ANOVA was performed, followed by Tukey’s honest significance difference (HSD) *post hoc* test or Kruskal-Wallis test was performed with Dunn’s multiple comparisons test. *p* < 0.05 was considered statistically significant.
