# Supplementary material for: ZO-1/Tjp1 and ZO-2/Tjp2 deletion in retinal pigment epithelium causes progressive retinal degeneration
Source: iScience. 2025 Oct 3;28(11):113673. doi: 10.1016/j.isci.2025.113673 (PMC12590551; doi:10.1016/j.isci.2025.113673)
Supplement: Document S1. Figures S1–S9 and Table S1 [file mmc1.pdf]

iScience, Volume *28*

Supplemental information

ZO-1/Tjp1 and ZO-2/Tjp2 deletion in retinal pigment epithelium causes progressive retinal degeneration

Safiah Mohamed Ali, Bhav Harshad Parikh, Queenie Shu Woon Tan, Barbara Hübner, Aleksandra N. Kozyrina, Animesh Banerjee, Jie Zhao, Debbie Goh, Hanumakumar Bogireddi, Sia Wey Yeo, Daniel Soo Lin Wong, Jianliang Xu, Kim Chi Tran, Zengping Liu, Yun-Zheng Le, Veluchamy Amutha Barathi, Kang Hao Cheong, Jacopo Di Russo, Alexander Ludwig, Walter Hunziker, and Xinyi Su

Supplementary Figure 1

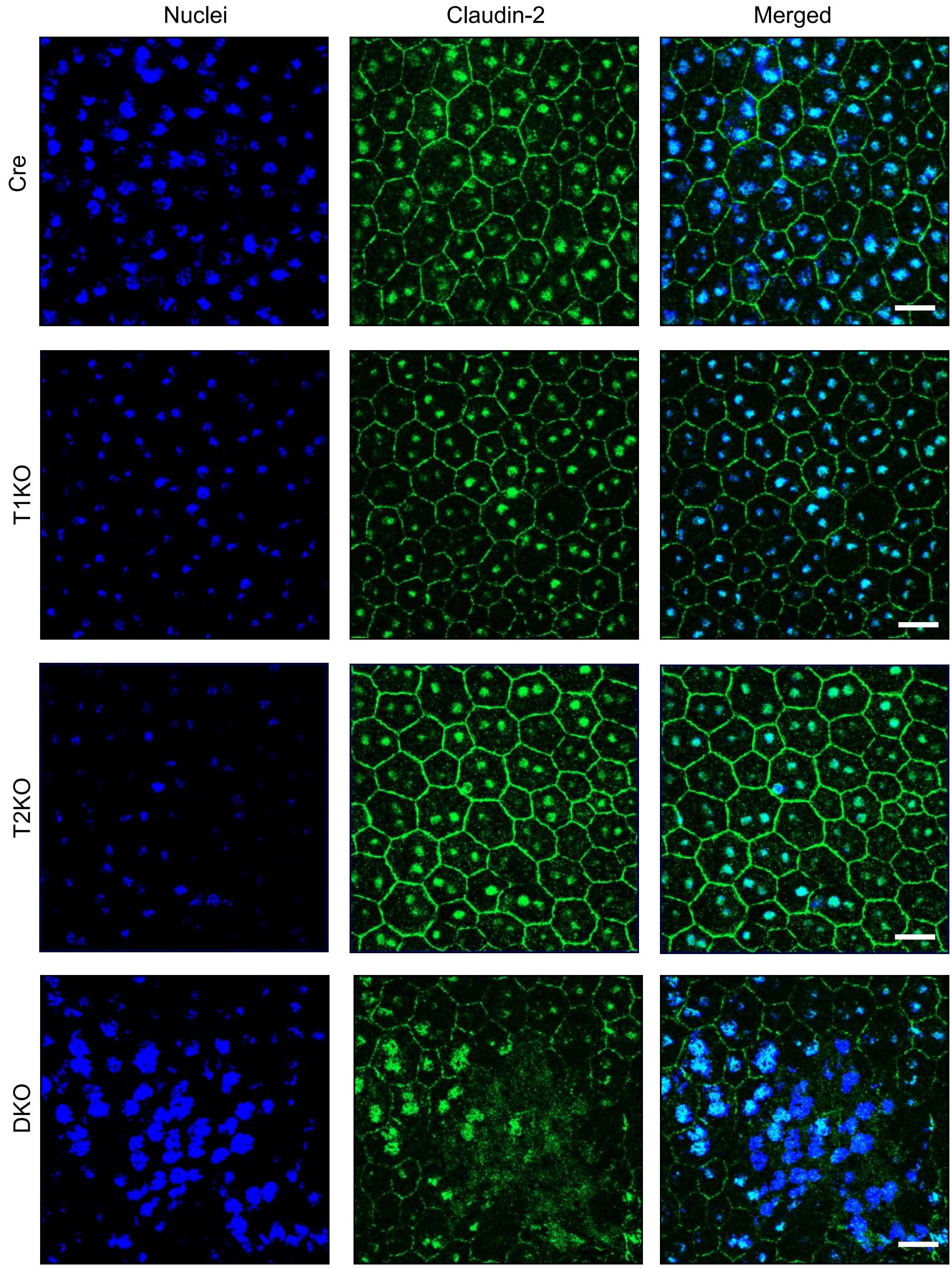

Fig. S1 | Expression and localization of Claudin-2 in T1KO, T2KO, DKO, and Cre Control at 1-month post Dox. Immunofluorescence was performed for Claudin-2. Junctional expression is observed in all 4 genotypes. DKO RPE showed patchy expression of Claudin-2, with complete loss in some regions. Scale bar, 20 μm.

Supplementary Figure 2

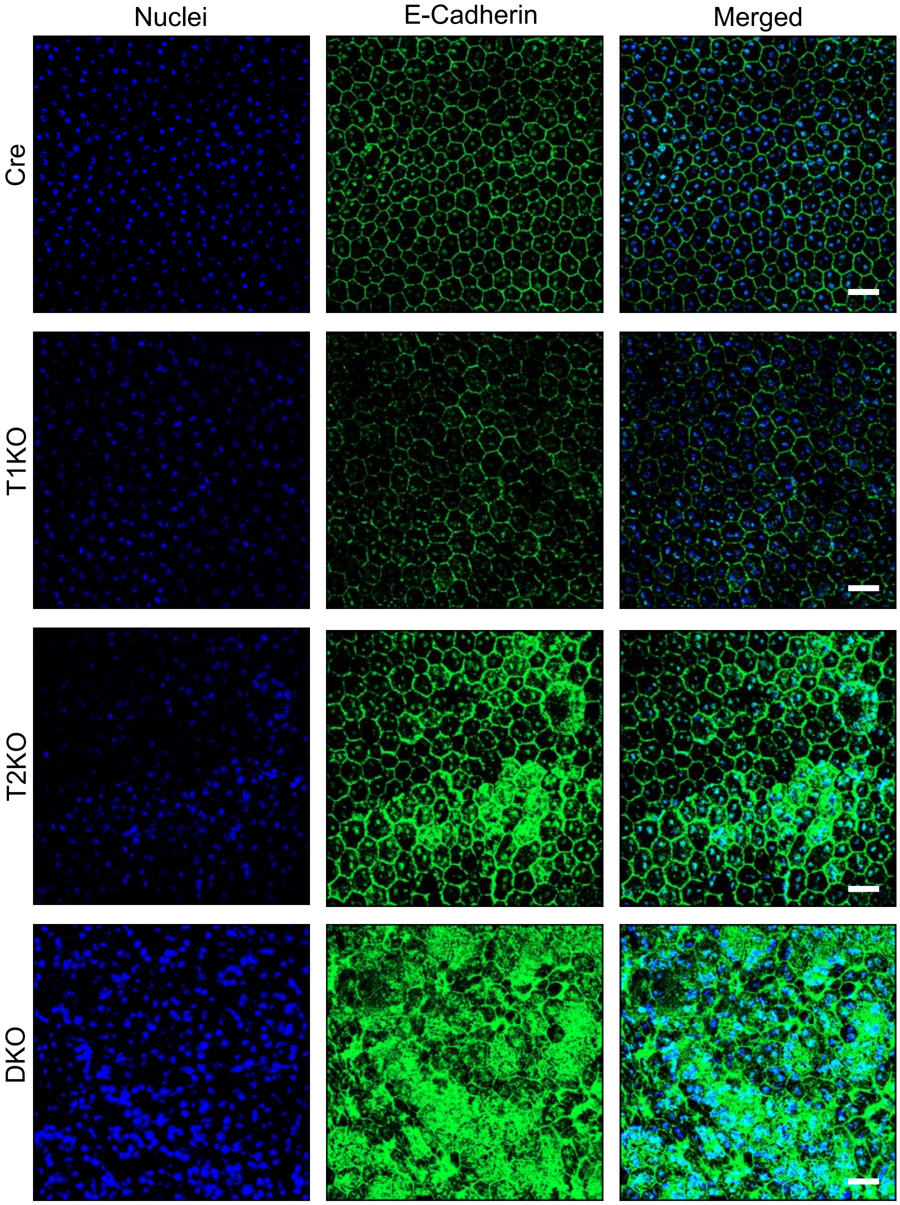

Fig. S2 | Cellular localization and protein expression of E-Cadherin in all four genotypes 1-month post Dox. IF analysis demonstrated junctional expression for E-Cadherin in all 4 genotypes. In addition, there was increased expression including cytoplasmic localization in T2KO, and more so in DKO RPE. Scale bar, 40 μm.

Supplementary Figure 3

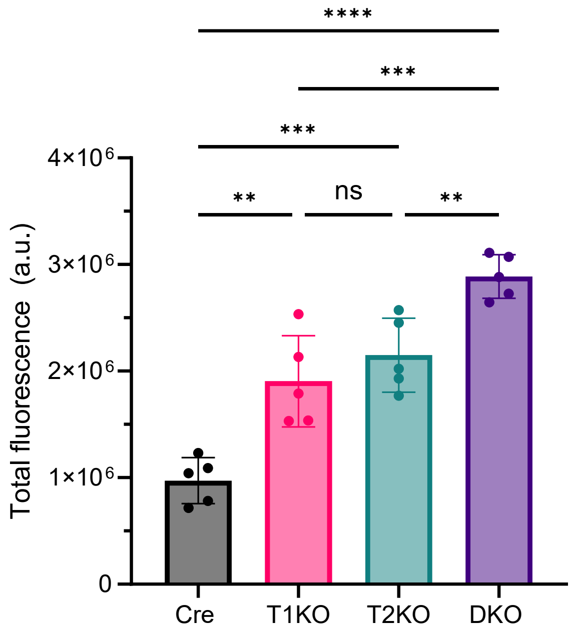

Fig. S3 | The fluorescence intensity from 40 kDa FITC-dextran leakage from choriocapillaris into the retina was quantified using ImageJ for all four mice genotypes. DKO retina showed the highest leakage of FITC-dextran. Statistical analysis was performed using one-way ANOVA, followed by Tukey's honest significance difference (HSD) *post hoc* test. (**) = $P < 0.01$, (***) = $P < 0.001$, (****) = $P < 0.0001$.

Supplementary Figure 4

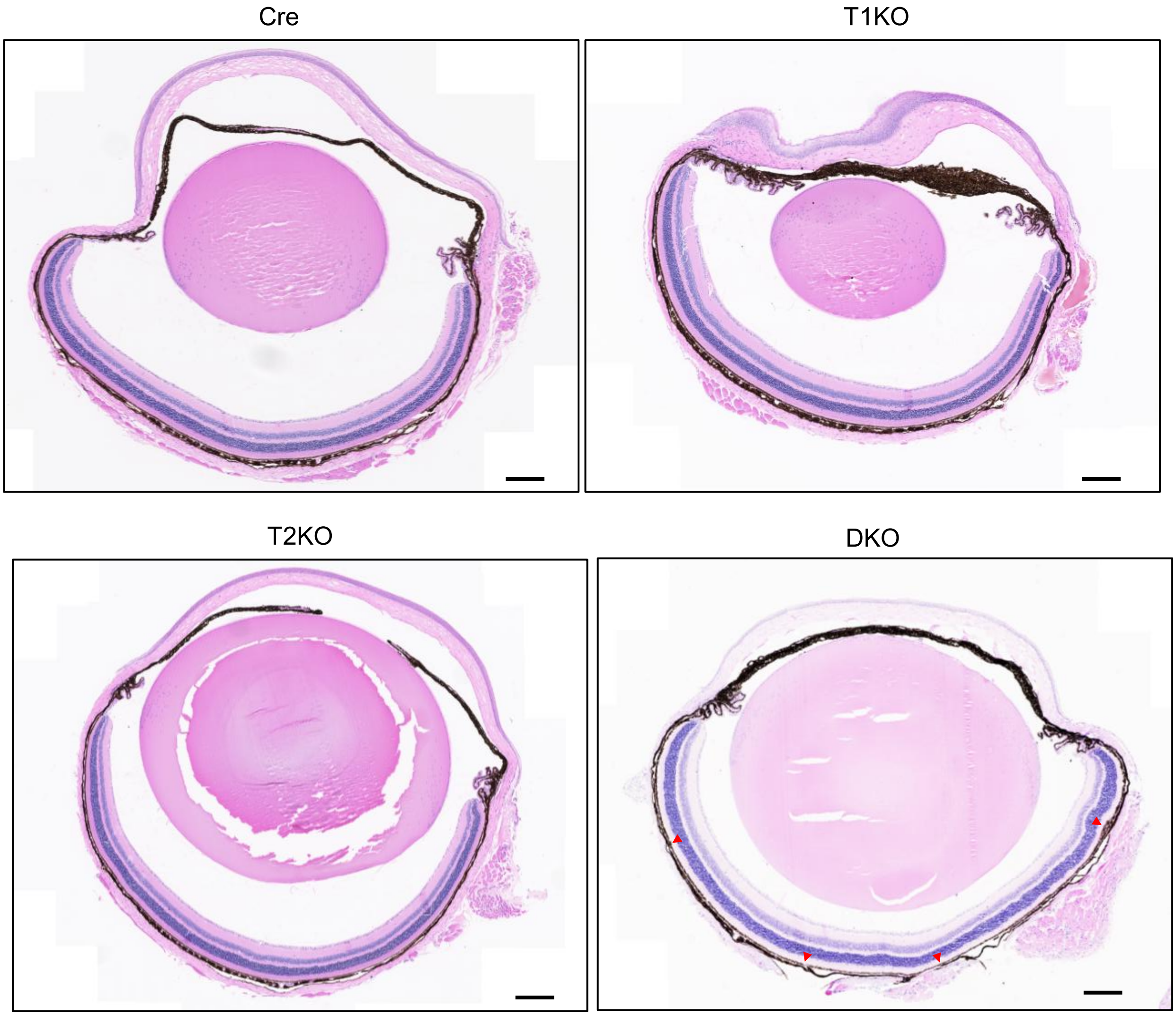

Fig. S4 | *Ex vivo* H&E stained eye cross-sections of all four genotypes at 1-month post-Dox. T1KO and T2KO have comparable retinal structure to Cre control. Whereas irregularities in the DKO RPE were observed, including decreased pigmentation and loss of monolayer epithelium was observed in marked regions (red arrows). Scale bar, 200 μm.

Supplementary Figure 5

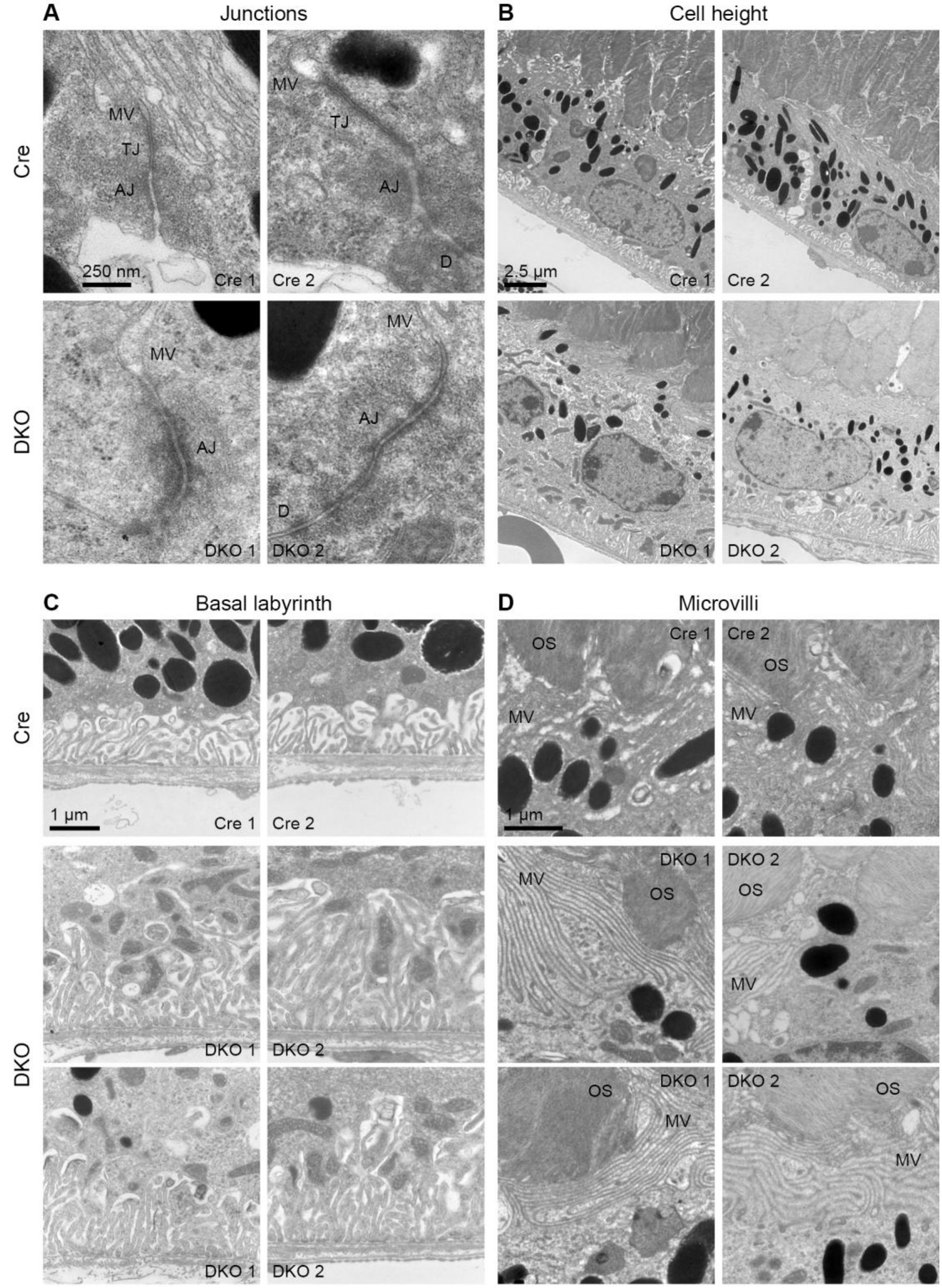

Fig. S5 | Ultrastructural changes in the DKO RPE 1-month post-Dox using transmission electron microscopy (TEM). Additional images corresponding to *Fig. 3C* showing the morphology of the RPE from two different sample pairs each for DKO (DKO 1/2; bottom rows) and Cre control (Cre 1/2; top row). Images in *Fig. 3C* are from sample pairs 1. (**A**) Tight junctions (TJ) are absent in DKO (bottom row); AJ = adherens junctions, D = desmosomes, MV = microvilli. (**B**) RPE cell height is increased in DKO (bottom row). (**C**) The basal labyrinth (BL) is increased in DKO (row two and three). (**D**) Microvilli (MV) appear less compact or reduced in DKO (row two and three); OS = Photoreceptor Outer Segments.

Supplementary Figure 6

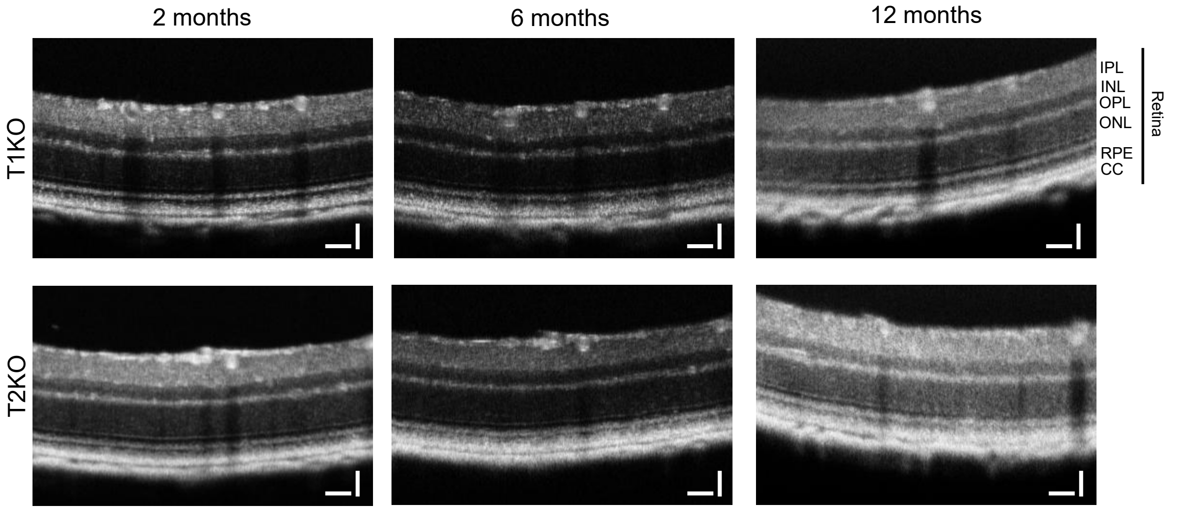

Fig. S6 | OCT images of T1KO and T2KO mice at various timepoints (Dox administrated on 1-month-old age). Retinal thickness (quantification in Fig. *5B, C*) remained comparable to Cre control for all time points. Scale bar, 50 µm. IPL = Inner Plexiform Layer. INL = Inner Nuclear Layer. OPL = Outer Plexiform Layer. ONL = Outer Nuclear Layer. RPE = Retinal Pigment Epithelium. CC = Choriocapillaris. OS = Outer Segments.

Supplementary Figure 7

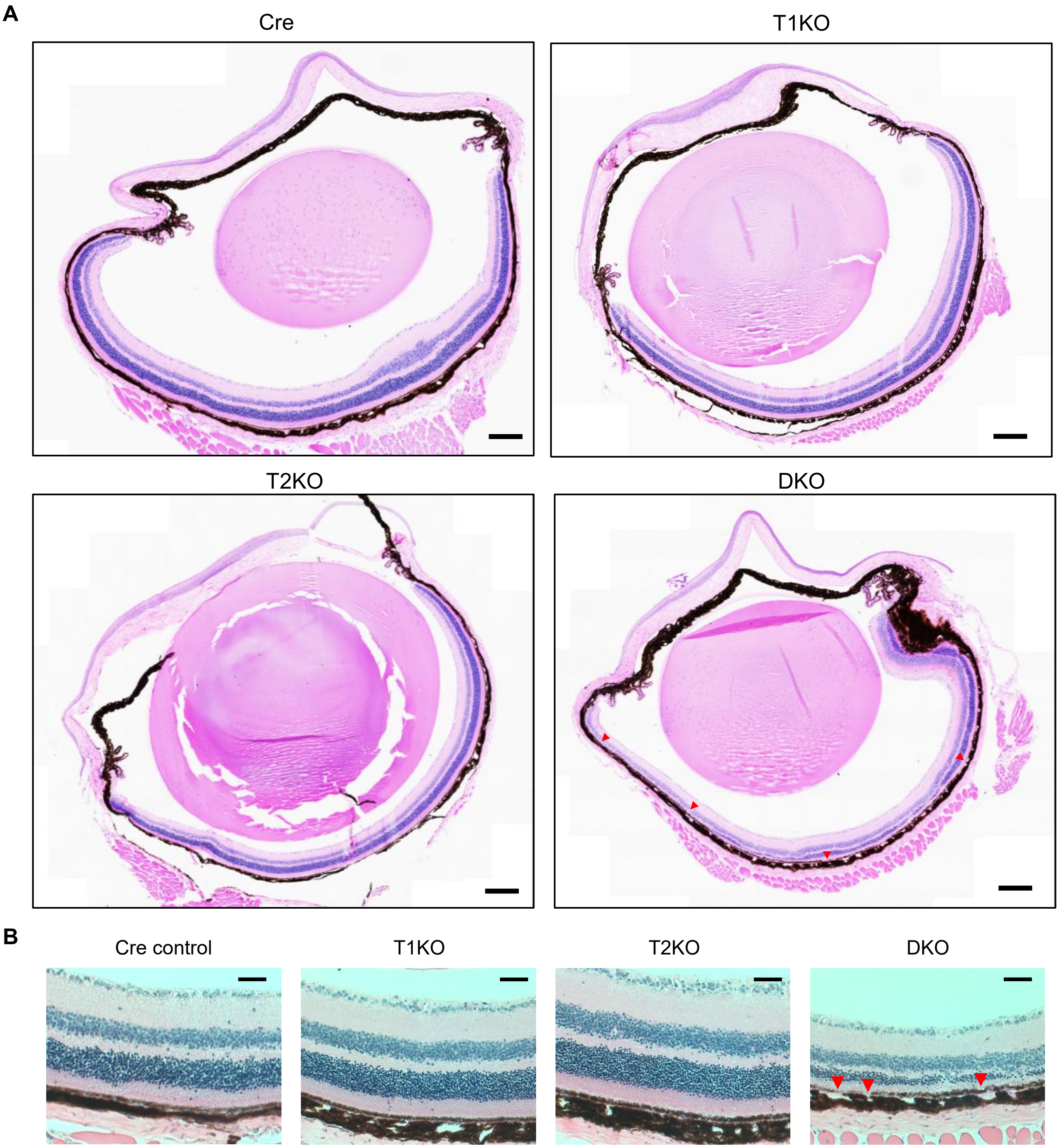

Fig. S7 | Ex *vivo* H&E stained cross-sections of all four genotypes at 12 months. (**A**) Full eye cross sections was visualized. T1KO and T2KO mice demonstrated normal retinal structure, comparable to the Cre control. Whereas, DKO demonstrated a severely thinned retina with irregularities and loss of pigmentation in the RPE monolayer (red arrowheads). Scale bar, 50 μm (**B**) Selected region of the retina was focussed on to visualize the loss of RPE cells, photoreceptor degeneration, and overall retinal thinning (red arrowheads) in DKO mice. Scale bar, 200 μm.

Supplementary Figure 8

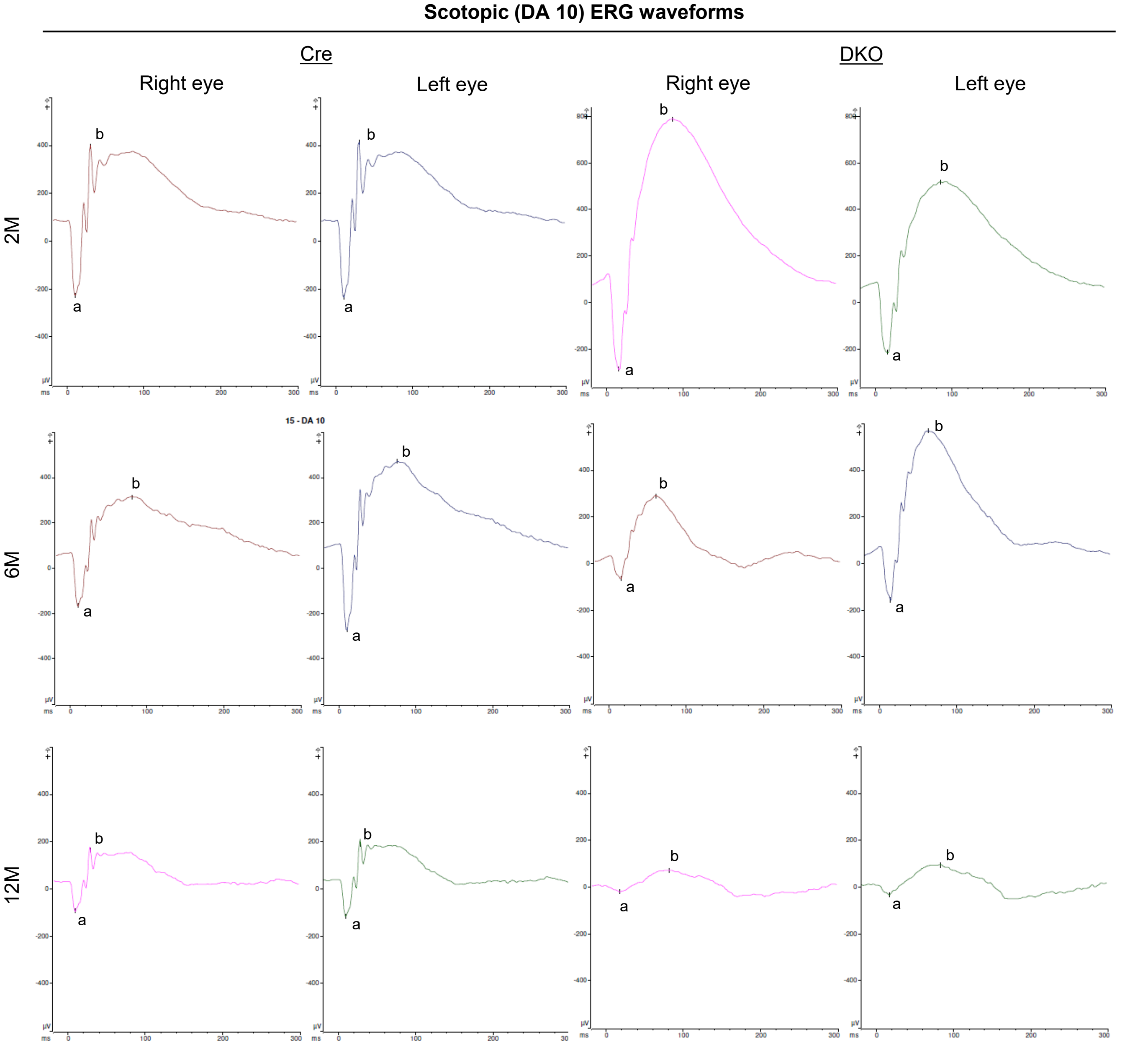

Fig. S8 | Representative scotopic (DA 10) ERG waveforms from both right and left eyes of a single Cre and DKO mouse at 2M, 6M and 12M. DA = dark adapted; a = a wave; b = b wave

Supplementary Figure 9

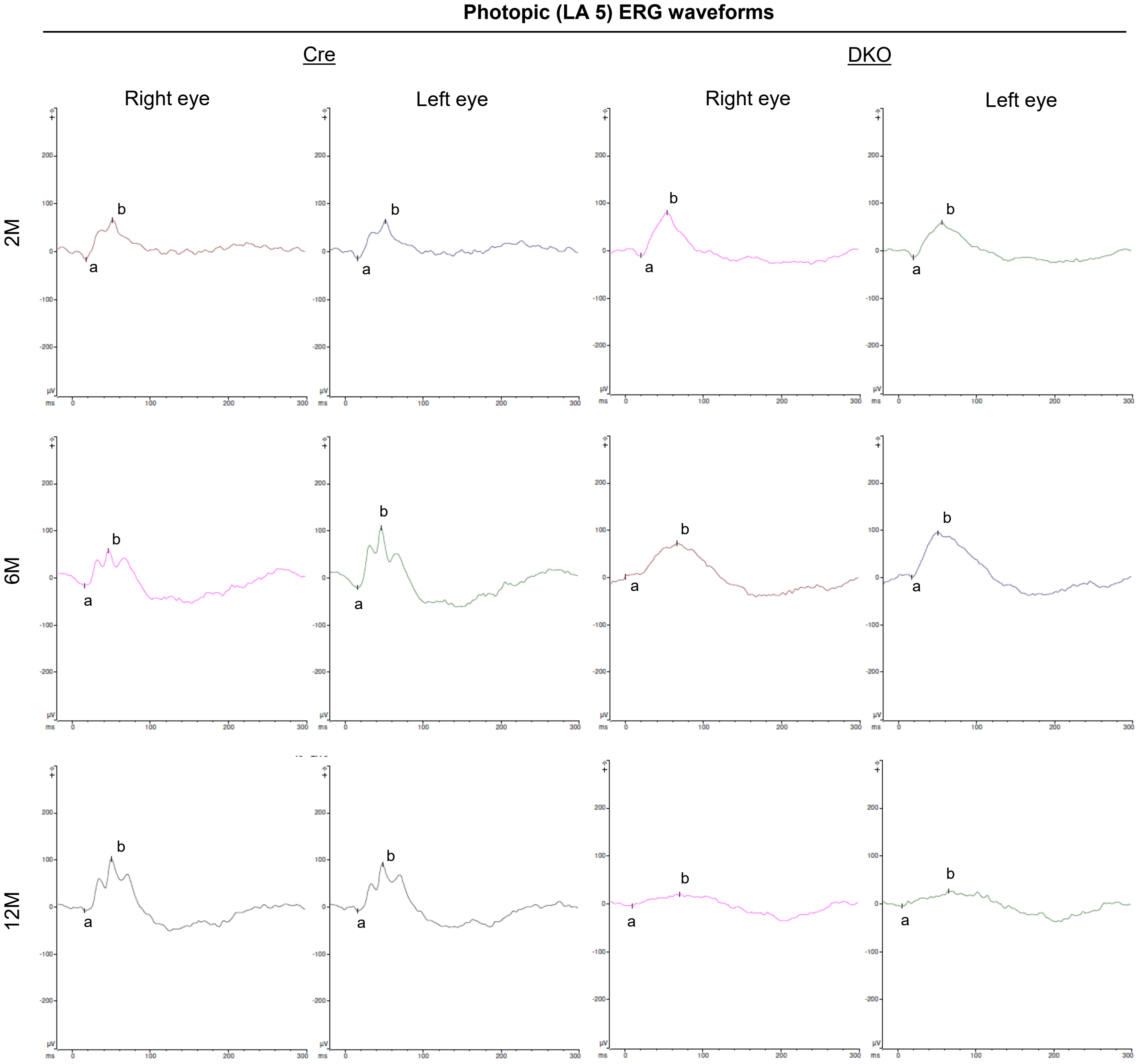

Fig. S9 | Representative photopic (LA 5) ERG waveforms from both eyes of a single Cre and DKO mouse at 2M, 6M and 12M. LA = light adapted; a = a wave; b = b wave

Supplementary Table 1 | Antibody list

Antibodies	Brand/ Manufacturer	Catalogue #	Dilution
Mouse anti-E-Cadherin	BD Biosciences	610181	1:100
Mouse anti-GAPDH	EMD Millipore	MAB374	1:2500
Mouse anti-PCNA	Cell Signaling Technology	2586	1:100
Mouse anti-RPE65	Abcam	ab13826	1:200
Rabbit anti-active Yap1 [EPR19812]	Abcam	ab205270	1:200
Rabbit anti-Claudin-2	Invitrogen	516100	1:100
Rabbit anti-Cytokeratin 18	Abcam	ab32118	1:100
Rabbit anti-Ezrin	Abcam	ab41672	1:100
Rabbit anti-RPE65	Abcam	ab231782	1:200
Rabbit anti-Vimentin	Abcam	ab15248	1:100
Rabbit anti-ZO1	Life Technologies	61-7300	1:100
Rabbit anti-ZO2	Life Technologies	38-9100	1:100
Rat anti-Nidogen	Millipore	MAB1946	1:100
Rat anti-ZO1	DSHB	R26.4C	1:50
